# Translocatome: a novel resource for the analysis of protein translocation between cellular organelles

**DOI:** 10.1093/nar/gky1044

**Published:** 2018-10-31

**Authors:** Péter Mendik, Levente Dobronyi, Ferenc Hári, Csaba Kerepesi, Leonardo Maia-Moço, Donát Buszlai, Peter Csermely, Daniel V Veres

**Affiliations:** 1 Department of Medical Chemistry, Semmelweis University, Budapest, Hungary; 2 Institute for Computer Science and Control (MTA SZTAKI), Hungarian Academy of Sciences, Budapest, Hungary; 3 Institute of Mathematics, Eötvös Loránd University, Budapest, Hungary; 4 Cancer Biology and Epigenetics Group, Research Center of Portuguese Oncology Institute of Porto, Portugal; 5 Turbine Ltd., Budapest, Hungary

## Abstract

Here we present Translocatome, the first dedicated database of human translocating proteins (URL: http://translocatome.linkgroup.hu). The core of the Translocatome database is the manually curated data set of 213 human translocating proteins listing the source of their experimental validation, several details of their translocation mechanism, their local compartmentalized interactome, as well as their involvement in signalling pathways and disease development. In addition, using the well-established and widely used gradient boosting machine learning tool, XGBoost, Translocatome provides translocation probability values for 13 066 human proteins identifying 1133 and 3268 high- and low-confidence translocating proteins, respectively. The database has user-friendly search options with a UniProt autocomplete quick search and advanced search for proteins filtered by their localization, UniProt identifiers, translocation likelihood or data complexity. Download options of search results, manually curated and predicted translocating protein sets are available on its website. The update of the database is helped by its manual curation framework and connection to the previously published ComPPI compartmentalized protein–protein interaction database (http://comppi.linkgroup.hu). As shown by the application examples of merlin (NF2) and tumor protein 63 (TP63) Translocatome allows a better comprehension of protein translocation as a systems biology phenomenon and can be used as a discovery-tool in the protein translocation field.

## INTRODUCTION

Subcellular localization of proteins is essential in spatial and temporal organisation of biological processes such as signalling pathways enabling their separation into organelles (1). Translocating proteins play a key role in the reconfiguration of cellular functions after environmental changes, as well as in embryonic or disease development. Different subcellular organelles have well characterized interactomes (2,3). With the advance of imaging techniques subcellular dynamics became a rapidly expanding research area (4,5). Restoring or affecting the cellular localization of disease-related proteins emerges as an efficient therapeutic method (6,7).

Protein translocation is a process which refers to the alteration of a given protein's subcellular localization. However, this phenomenon has no unified definition, and the word ‘translocation’ may also refer to gene translocation or RNA translocation at the ribosome. In this work we define protein translocation as a systems biology phenomenon, which refers to the regulated movement of a protein of a given post-translational state between subcellular compartments. Translocation changes the interaction partners and leads to altered function(s) of translocating proteins. There are certain processes (such as co-translational, post-translational delivery-type, cell division-induced, downregulation- or passive diffusion-related phenomena; for their detailed description see Supplementary Texts S1 and S2) that may change the localization of a protein, but to increase the focus and clarity of our database we did not consider them as translocation.

There are widely used protein databases that contain information on protein translocation, e.g. the MoonProt (8) or UniProt (9) databases. However, these databases are not dedicated collections of translocating proteins. Here we present Translocatome, which is a manually curated database of 213 human translocating proteins with extensive information on their translocation. Moreover, Translocatome contains 13 066 human proteins with predicted likelihood of translocation. With the help of the well-established and widely used gradient boosting machine learning tool, XGBoost (10–12) we predicted 1133 high-confidence translocating proteins. In addition, Translocatome contains 3268 and 8665 low-confidence and non-translocating proteins, respectively. To train the XGBoost algorithm, we also created a manually curated set of 139 non-translocating proteins as part of the database. In summary, Translocatome is a novel, dedicated database of human translocating proteins including their interaction partners in the different subcellular localizations. This database contributes to a better understanding of protein translocation as a systems biology phenomenon and facilitates further discoveries of translocating proteins. As translocating proteins are already targeted pharmaceutically (6,7) new findings in this field may lead to better therapeutic options.

## DESCRIPTION OF THE DATABASE

### Overview of translocatome

**Figure 1.**
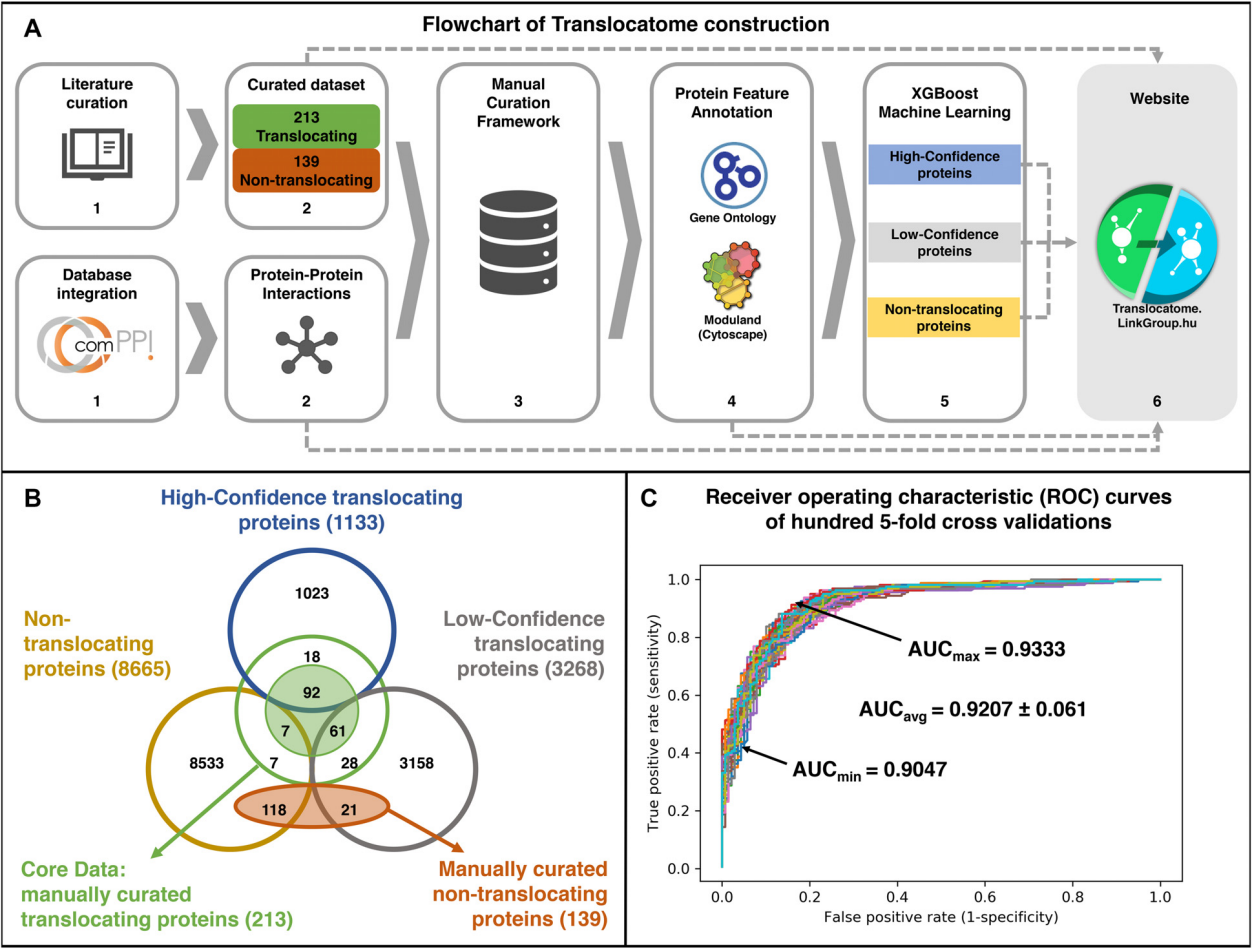
The structure of the Translocatome database and performance of the XGBoost machine learning prediction method. (**A**) Schematic flowchart of the Translocatome database construction process highlighting 6 major steps. The panel shows the main input sources of the Translocatome are manual curation of peer reviewed articles and the ComPPI database (http://comppi.linkgroup.hu; 3). In the manual curation process we recorded the source of experimental validation, several details of translocation mechanism, the local compartmentalized interactome, as well as the involvement in signalling pathways and disease development **(1)**. This extensive manual curation resulted in a set of 213 translocating and another set of 139 non-translocating human proteins. To incorporate our data into a Protein-Protein Interaction (PPI) network we imported the PPI of 13 066 ComPPI (3) human proteins with their 151 889 interactions **(2)**. The Manual Curation Framework (MCF) is a user-friendly interface where the data of the Translocatome database is stored and after registration users from all over the world can log in to modify and update its data, which is published as part of the Translocatome database after expert cross-check **(3)**. To enable the prediction of translocating proteins we annotated each protein in our database with Gene Ontology (13,14) functional and ComPPI-derived interactome (3) topological properties **(4)**. The XGBoost machine learning algorithm (10–12) classified the 13 066 human proteins into three sets: high-, low-confidence translocating proteins and non-translocating proteins **(5)**. On the http://translocatome.linkgroup.hu website the whole dataset is available for searching and downloading purposes freely and without registration. Translocatome can be updated by the community-based Manual Curation Framework. Moreover, Translocatome is linked to the ComPPI database (3) so in the case of its update Translocatome can be also updated **(6)**. (**B**) Structure of the Translocatome database. As shown by a Venn-diagram the database consists of the Core Data of 213 manually curated translocating proteins (available here: http://translocatome.linkgroup.hu/coredata), which are extended by 1133 and 3268 high- and low-confidence translocating proteins, respectively. Green and red filled circles represent the positive and negative training sets, respectively. Core Data and positive learning set differ, since the latter does not contain the 53 proteins showing translocation exclusively under pathological conditions (such as cancer). (**C**) Performance of the widely-used XGBoost machine learning method (10–12) on the final feature set. Each of the 100 different receiver operating characteristic (ROC) curves belong to a different 5 fold cross-validation run on the training set (containing 160 physiologically translocating and 139 non-translocating proteins). In the runs the XGBoost machine learning method used the final feature set (see Table 1) selected earlier as described in the main text and Supplementary Text S6. The minimal, maximal and average area under the curve (AUC) were 0.9047, 0.9333 and 0.9207 (±0.0061 standard deviation), respectively.

Translocatome is the first database that collects manually curated human translocating proteins including their interacting partners in the localizations involved, translocation mechanism (including protein structure details if available), type of experimental evidence, affected signalling pathway(s) and pathological properties. The core of the Translocatome database is the 213 manually curated human translocating proteins (http://translocatome.linkgroup.hu/coredata) which were all collected based on related publications containing experimental evidence. Altogether Translocatome contains 13 066 human proteins, which were selected from the compartmentalized protein–protein interaction database (3; ComPPI http://comppi.linkgroup.hu, downloaded on 20 July 2018) using the inclusion criterion that every protein needed to have at least one experimentally validated subcellular localization. By the application of the well-established gradient boosting machine learning tool, XGBoost (10–12) we predicted 1133 high-confidence translocating proteins. All the 13 066 human proteins were characterized by their translocation likelihood named as Translocation Evidence Score (TES) calculated by the XGBoost machine learning algorithm (Figure 1). Various search and download options make it possible for users to process these data according to their goals.

### Database content

The core data of Translocatome is the extensively curated set of 213 human translocating proteins (see Core Data at the website: http://translocatome.linkgroup.hu/coredata). With the manual curation process involving the judgement of 3 independent experts we aimed to collect detailed and experimentally validated information about every entry extracted from peer reviewed publications (for the details of the manual curation process see Supplementary Text S3, Supplementary Table S1 and Supplementary Figure S1). For each of the 213 manually curated translocating proteins we collected the available subset of the following data:

- name set, gene name and UniProt (9) accession number and link,
- PubMed ID(s) and link(s) to peer-reviewed article(s) describing the experimental evidence of translocation,
- initial and target localizations of the translocating protein,
- interacting partners and biological functions (both in the initial and target compartments),
- translocation mechanism,
- the used detection method,
- protein structural information on translocation mechanism,
- disease group, exact disease involved and pathological role,
- signalling pathways affected.

We used the UniProt naming convention (9) for protein identification, Gene Ontology terms (13,14) for localization/biological process identification and the KEGG naming convention (15) for the standardization of signalling pathways. Following the logic of our previously published compartmentalized protein–protein interaction database (ComPPI, 3) every protein was annotated with one of six major cellular localizations (cytoplasm, extracellular space, mitochondria, nucleus, membrane or secretory-pathway). If there was more precise localization information available it was included as a minor localization. All 213 manually curated translocating proteins are characterized by a Data Complexity Score (DCS) as described later in detail, which makes it easier to assess the amount of information associated with each protein. 53 of the manually curated proteins showed translocation exclusively under pathological conditions (such as cancer). Therefore, we used the remaining 160 physiologically translocating proteins as a positive training set (Supplementary Table S2) for the widely used XGBoost machine learning algorithm (10–12).

We also collected a manually curated negative dataset of 139 human non-translocating proteins, each one classified as a protein **(a)** with experimentally proved diffuse, multi-compartmental distribution, **(b)** with exclusive single-compartment localization, **(c)** docked to DNA/RNA, **(d)** embedded in membranes or **(e)** attached to the cytoskeleton (for additional details, see Supplementary Text S4). These 139 proteins were used as a negative training set (Supplementary Table S3) for the application of the XGBoost machine learning algorithm (10–12). For a detailed description of our database structure, see Figure 1A and B.

Altogether Translocatome contains 13 066 human proteins having at least one experimentally validated localization as described in our in house developed compartmentalized protein–protein interaction database (ComPPI, 3). From the ComPPI database we also imported the interactome of these human proteins having 151 889 interactions. The translocation likelihood of all the 13 066 proteins is characterized by a Translocation Evidence Score (TES) as described later in detail. The translocation likelihood was calculated by the XGBoost machine learning algorithm (10–12) as detailed in the next Section.

### The XGBoost machine learning algorithm-based prediction of translocating proteins

The machine learning procedure followed the general methodology of supervised machine learning workflow: data collection, feature extraction, feature selection, classification, training, testing and interpretation. For each step we applied an existing, well-characterized approach. Data collection and feature extraction were based on established procedures as described below. For all additional steps we applied the well-established, widely used gradient boosting-type (10) machine learning tool, XGBoost (11). XGBoost was successfully applied in hundreds of recent studies to predict, e.g. host-pathogen protein–protein interactions (16), microRNA disease association (17) and DNA methylation (18). Several studies including our own previous paper showed that XGBoost gives the best performance if compared with a number of known machine learning methods (see e.g. Refs. 12, 16 and 18).

To train the XGBoost method first we annotated each of the 13 066 proteins of the Translocatome database with their relevant Gene Ontology (GO, 13,14) cellular component, biological process and molecular function terms also including their ancestors. This resulted in 21 020 annotated GO terms total (all details of the methodology are available here: https://github.com/kerepesi/translocatome_ml). The process was based on our previous work (12), for its details please see Supplementary Text S5.

Next, each of the 13 066 proteins were annotated with their degree and bridgeness in the compartmentalized protein–protein interaction database (ComPPI, 3) derived human interactome containing 151 889 interactions. Degree (the number of human interactome neighbours) was included, since the 213 manually curated translocating proteins showed a significantly higher degree than that of the 139 manually curated non-translocating proteins or the average (Supplementary Figure S2). This is not surprising since translocating proteins often have a central role in regulation behaving as interactome hubs. Similarly, translocating proteins often connect interactome modules (large protein mega-complexes), thus act as bridges. Indeed, the 213 manually curated translocating proteins had significantly higher bridgeness values than that of the 139 manually curated non-translocating proteins or the average (Supplementary Figure S2). Degree and bridgeness values were calculated using the CytoScape network analyser program (19) and its ModuLand plug-in (20), respectively. GO terms, degree and bridgeness formed the feature sets selected by the XGBoost machine learning method.

Since the human interactome (3) we used for the calculation of degree and bridgeness did not contain interactions observed in pathological conditions, we excluded those 53 of the manually curated proteins from the positive training set of the XGBoost algorithm, which showed translocation exclusively under pathological conditions (such as cancer). The remaining 160 manually curated proteins were used as the positive training set (Supplementary Table S2).

Following the methodology of several XGBoost studies (11,16–18) including our previously published work (12) we evaluated the XGBoost-selected feature sets by 5-fold cross-validation, and we evaluated their predictive power by the area under the curve of the receiver operating characteristic curve (ROC AUC or shortly AUC, 21). 5-fold cross-validation is a widely used method where the training data is split into five random parts and four parts are used to train the XGBoost machine learning tool and the prediction of the fifth part is evaluated. For every feature set, we repeated this process 100 times. We selected those GO features which had a feature important value (produced by the XGBoost program) greater than 0.02. With this generally applied XGBoost procedure we reached an average AUC of 0.916 (±0.0046 standard deviation) with only 15 GO features left from the initial 21 020 (see Table 1). We continued feature selection by adding the two interactome-derived features degree and bridgeness using the giant component of the ComPPI-derived human protein–protein interaction network (3). In these calculations the giant component of the interactome was used which did not contain 9 proteins of the total. The inclusion of the two network-related features produced an average AUC of 0.9207 (±0.0056 standard deviation), showing a further increase from the average AUC of 0.916 and implying a high performance. We show the ROC curves of 100 five-fold cross-validation runs of the final feature set on Figure 1C having a minimal, average and maximal AUC of 0.9047, 0.9207 and 0.9333, respectively. As shown on Supplementary Figure S3 both precision-recall and Matthews correlation coefficient curves also showed a high performance of the learning process. For more details of the generally applied machine learning procedure, see Supplementary Text S6. All data of the procedure are available at https://github.com/kerepesi/translocatome_ml along with codes to reproduce the results.

**Table 1.** The feature set identified as best predictor by the XGBoost machine learning algorithm

Gene Ontology process (GO term name) or interactome feature	Importance	Short biological explanation
**Parameters having a positive predictive value**		
*animal organ morphogenesis (GO:0009887)*	2.68	Morphogenesis and other developmental processes are mostly regulated through complex networks of transcription factors, where translocation is often involved as a regulation step (29).
*regulation of carbohydrate metabolic process (GO:0006109)*	1.53	Quite some metabolic enzymes also function as protein kinases and translocate between cellular compartments playing a role e.g. in carcinogenesis (30).
*cytoplasm (GO:0005737)*	1.35	Large cellular compartments are often associated with proteins that translocate. Nucleo-cytoplasmic translocations play a key role in the regulation of transcription factors (29).
*nuclear part (GO:0044428)*	1.12	
*negative regulation of cellular process (GO:0048523)*	1.12	Negative regulatory mechanisms are frequently exerted by translocating proteins such as e.g. PTEN (31) or transcription factors.
*plasma membrane part (GO:0044459)*	0.70	Large cellular compartments are often associated with proteins that translocate. Cytosol-membrane translocations play a key role in the regulation of signalling pathways (32).
*extracellular region (GO:0005576)*	0.65	
*cytosol (GO:0005829)*	0.57	
*spliceosomal complex (GO:0005681)*	0.23	The spliceosome is constituted by snRNPs translocating from the cytoplasm. Some spliceosome components are also involved in mRNA export (33,34).
**Parameters having a negative predictive value**		
*bridgeness value is lower than 0.000292 (bridgeness lower than 0.000292)*	−0.36	Translocating proteins often bridge the two interactome modules (large protein complexes) of their two localizations. Therefore, their bridgeness values tend to be high (20 and Supplementary Figure S1).
*degree is smaller than 62.5 (degree lower than 62.5)*	−0.50	A reasonably high number of interaction partners often indicates a role in regulation and signal transduction. Many of these proteins are ‘date-hubs’, which may undergo a translocation process. Nevertheless, too many partners could be a characteristics of a multi compartmental housekeeping protein (35 and Supplementary Figure S1).
*degree is smaller than 14.5 (degree lower than 14.5)*	−0.54	
*negative regulation of intracellular signal transduction (GO:1902532)*	−0.61	If the translocation process becomes inhibited, it may often prevent signal transduction. Inhibition often occurs via sequestration by large protein complexes which usually have only one localization (36).
*myeloid cell differentiation (GO:0030099)*	−0.74	Cell adhesion and membrane bound proteins play an important role in myleoid cell differentiation (37,38). Both protein categories are typically non-translocating proteins, which may over-compensate the role of translocating transcription factors in this process.
*intrinsic component of membrane (GO:0031224)*	−0.82	Intrinsic membrane components predominantly do not translocate to other major localizations.
*system process (GO:0003008)*	−0.91	A wide variety of proteins exert their system level biological functions (e.g. secretion of molecules) in a non-translocating manner: cell membrane channels, actin, myosin, etc.
*single organismal cell-cell adhesion (GO:0016337)*	−1.06	Cell adhesion proteins usually have a strictly limited location in the plasma membrane
*bridgeness value is lower than 2.5e-06 (bridgness lower than 2.5e-06)*	−1.10	Translocating proteins often bridge the two interactome modules (large protein complexes) of their two localizations. Therefore, their bridgeness values tend to be high (20 and Supplementary Figure S1).
*protein complex (GO:0043234)*	−1.24	Proteins often fulfil their roles in large protein mega-complexes. These complexes may assist for other proteins to translocate, but their own components do not translocate.

Features selected by the XGBoost machine learning algorithm (10–12) can be human protein–protein interaction network-related (3) or GO term-related (13,14), as listed in the first column. XGBoost assigns each feature with an importance score (as shown in the third column) which was calculated as the leaf-scores of the one-depth trees of the best XGBoost model. In the fourth column there is a short (and most of the time, very partial) explanation to explain why these features may become selected by the XGBoost machine learning process as best predictors of protein translocation including some key references supporting the explanations.

The feature set of the XGBoost model with the best AUC value is shown on Table 1. Features with positive importance values increase the probability of translocation. These are Gene Ontology features from each main GO category (cellular components, biological processes and molecular functions), which are often associated with protein translocation as described in Table 1 in detail. If a feature has a negative importance value, then it decreases the probability of translocation. Two categories of low degree and low bridgeness values each, as well as six GO-terms negatively associated with protein translocation are listed among these negative features. Using the feature set shown on Table 1 we calculated the Translocation Evidence Score characterizing the translocation probability of each of the 13 066 proteins in the Translocatome database as described in the next section.

### Data complexity and translocation evidence scores

#### Data complexity score

To provide an easy assessment of the information available of a manually curated protein we developed the Data Complexity Score (DCS). DCS varies between 0 and 1, having increasing values if the protein has more curated data. The score is calculated and normalized after weighting all the available data, where those related to translocation have a higher weight (please find the detailed calculation process in Supplementary Text S7). Therefore, DCS is not only shows the quantity but also the relevance of the available data. In addition DCS indicates which entries may require further curation.

#### Translocation evidence score

The XGBoost machine learning method gave every protein of the Translocatome database a Translocation Evidence Score (TES) that is proportional with the translocation probability of the given protein. For each protein we computed TES using Equation (1)

(1) \begin{equation*}\sum\nolimits_{i\ = \ 1}^n {{w_i}{x_i}} ,\end{equation*}

where *wi* is the importance value of the *i* th feature of the model (see *i*th row of Table 1). The importance value was calculated as described in the legend of Table 1. *x = 1*, if the given feature is true for that protein and *x = 0*, if it is false (*n* is the number of features of the model; here *n* = 19). TES values were rescaled to the interval [0,1] by min–max normalization using Equation (2)

(2) \begin{equation*}x^\prime = \frac{{x - {x_{min}}}}{{{x_{max}} - {x_{min}}}}\ ,\end{equation*}

The larger the TES value, the greater the probability of translocation. As a numerical example, suppose that ‘protein A’ has 20 neighbours (degree) in the human interactome and its UniProt record contains only two GO terms, ‘animal organ morphogenesis’, and ’cytoplasm’. Then the predicted translocation evidence score of ‘protein A’ is −0.497 + 2.675 + 1.353 = 3.531. The value is then normalized using Equation (2). For each of the 13 066 proteins, the respective TES scores can be found both in the search results and in the downloadable datasets.

The Translocation Evidence Score gave the possibility to define a cut-off value, below which proteins were considered as non-translocating. To define this cut-off value, we used the widely used measure of a test's accuracy, the F1 score (also called as F-measure, 22) that measures the performance of a binary classification being a harmonic average of precision and recall (also called as sensitivity). Supplementary Figure S4 shows recall, fallout, precision and the F1 score at different threshold values and illustrates the distribution of the TES values. The F1 score reached its maximum at the threshold of 0.4487, which gives a straightforward cut-off value for translocation probability. Thus proteins having lower TES values than 0.4487 were considered as non-translocating (for more details see Supplementary Texts S6 and S8). In order to give an assessment of potential false positive predictions we also defined a higher TES cut-off value separating low- and high-confidence translocating proteins. We set this value as 0.6167, since above this threshold there were not any negative set proteins. We assume that the probability of false positive predictions is low above this threshold value. Low-confidence translocating proteins, which have a translocation evidence score (TES) between the two threshold values are presumably translocating but they need further validation.

The two Translocation Evidence Score cut-off values separated our original 13 066 human proteins to three classes: **(a)** 1133 high-confidence translocating proteins having a TES value higher than 0.6167; **(b)** 3268 low-confidence translocating proteins having a TES value between 0.6167 and 0.4487, as well as **(c)** the residual 8665 proteins having a TES value lower than 0.4487, which were considered as non-translocating (Figure 1B).

### Search, download options and output

As part of the user-friendly interface, various search functions were developed. We provide an easy to use quick search function (with UniProt AC autocompletion) which can be used to find protein families or a given protein. The advanced search option creates the possibility to search for more elaborate sets of proteins filtered by their localization, UniProt identifiers, Translocation Evidence Score or Data Complexity Score. The web interface provides eight pre-defined protein sets as download options covering **(i)** 213 manually curated translocating proteins, **(ii)** 160 physiologically translocating manually curated proteins (the positive training set), **(iii)** manually curated non-translocating proteins (the negative training set), **(iv–vi)** high-, low-confidence and non-translocating protein sets, as well as **(vii)** the whole protein set and **(viii)** its protein–protein interaction network. These sets of proteins can be downloaded in a comma separated .csv format. Besides these pre-defined sets users can also download the results of their search queries as a tabulator separated file (.tsv, see the technical parameters in the ‘Design and implementation’ section). Examples and explanations of the output formats are available in Supplementary Figures S5–S7.

### Design and implementation

To allow the development of the Translocatome database as a community effort a manual curation framework (MCF) was designed. MCF uses the same MongoDB database as the Translocatome site, with a user interface developed in the Ruby on Rails 4.2 (https://rubyonrails.org) framework. The MCF website follows the hierarchical model-view-controller design pattern to ensure the separation of the data layer from the business logic and the user interface. The MCF stores all the data of the Translocatome and provides them to the front-end of the Translocatome website after expert review. To ease usability an end-user documentation is available as tutorials, detailed descriptions and location-specific tooltips in the HELP menu on the site (http://translocatome.linkgroup.hu/help). Further details of design and implementation of the database are summarized in Supplementary Text S9.

### Application examples

The Translocatome database is the only current dedicated collection of human translocating proteins. With its Translocation Evidence Score (TES) for 13 066 proteins it helps the identification and experimental validation of novel translocating proteins. To demonstrate the prediction efficiency of Translocatome we assessed the first 40 proteins with the highest TES values. Table 2. shows the list of the best performing 25 proteins. They fall into four categories: **(A)** were already included in the manually curated 213 translocating protein set (12 proteins: PTEN, PTK2, FOXO3, GMNN, ATF2, MAPK1, GLI3, HRAS, AR, SMAD3, SMAD2 and HSP90AB1); **(B)** were previously shown to be translocating proteins but have not appeared in our Core Data of 213 proteins collected from keyword-based searches (11 proteins: NF2, TULP3, SNCA, FGFR2, MTOR, GSK3B, EIF6, HDAC1, CARM1, CUL1 and RARB; see Supplementary Table S4); **(C)** have not been described as translocating proteins yet, but from the literature we can conclude that their translocation is probable (one protein: TP63); **(D)** there is no information in the literature about their translocation (one protein: PRKRA). Proteins of categories (C) and (D) are good candidates for further experimental studies verifying their translocation.

**Table 2.** List of the first 25 proteins having the highest Translocation Evidence Score

UniProt ID	Gene names	Protein names	Translocation evidence score	Group	Summary
**P60484**	PTEN	**Phosphatidylinositol 3,4,5-trisphosphate 3-phosphatase and dual-specificity protein phosphatase**	1.0000	A	PTEN translocates to the nucleus from the cytoplasm in response to oxidative stress
**P35240**	NF2	**Merlin**	0.9807	B	Dephosphorylated merlin translocates to the nucleus (23)
**O75386**	TULP3	**Tubby-related protein 3**	0.9802	B	Membrane association with PIP2 anchors Tub to sequester TULP3 from transport to the nucleus. It also translocates from the plasma membrane to the nucleus upon activation of guanine nucleotide-binding protein G(q) subunit alpha (39).
**Q05397**	PTK2	**Focal adhesion kinase 1 (FADK 1)**	0.9798	A	Retinoid acid induced nuclear FAK translocation leads to a reduced cellular adhesion
**P37840**	SNCA	**Alpha-synuclein**	0.9743	B	Mitochondrial translocation occurs rapidly under as a result of pH changes during oxidative or metabolic stress (40)
**O43524**	FOXO3	**Forkhead box protein O3**	0.9740	A	Dephosphorylated cytoplasmic Foxo is unidirectionally translocated out of the cytoplasm by the nuclear localization signal and Ran GTPase driven nuclear import system.
**O75496**	GMNN	**Geminin**	0.9740	A	Geminin is excluded from the nucleus during part of the G1 phase and at the transition from G0 to G1.
**P21802**	FGFR2	**Fibroblast growth factor receptor 2 (FGFR-2)**	0.9703	B	Under PGF(2alpha) stimulation, FGF-2 and FGFR2 proteins accumulate near the nuclear envelope and co-localize in the nucleus of Py1a cells (41).
**P15336**	ATF2	**Cyclic AMP-dependent transcription factor ATF-2**	0.9677	A	Some drugs as paclitaxel or vemurafenib are inducers of ATF-2 translocation.
**P42345**	MTOR	**Serine/threonine-protein kinase mTOR**	0.9635	B	Long-term treatment with rapamycin triggers dephosphorylation and cytoplasmic translocation of nuclear rictor and sin1 accompanied by inhibition of mTORC2 assembly (42).
**P28482**	MAPK1	**Mitogen-activated protein kinase 1**	0.9480	A	MAPK1 (ERK2) translocates to the nucleus and mitochondria.
**P49841**	GSK3B	**Glycogen synthase kinase-3 beta**	0.9462	B	GSK3 translocated to the plasma membrane, along with AXIN, upon Wnt stimulation (43).
**O75569**	PRKRA	**Interferon-inducible double-stranded RNA-dependent protein kinase activator A**	0.9439	D	There is no information in the literature about the translocation of this protein
**P10071**	GLI3	**Transcriptional activator GLI3**	0.9439	A	Translocates after interaction with ZIC1.
**P01112**	HRAS	**GTPase Hras**	0.9224	A	Several pathological conditions such as exogenous hyperoxia induce Ras translocation from cytosol to the membrane.
**P56537**	EIF6	**Eukaryotic translation initiation factor 6**	0.9208	B	Increase in intracellular concentration of calcium leads to rapid translocation of eIF6 from the cytoplasm to the nucleus, an event that can be blocked by specific calcineurin inhibitors, such as cyclosporin A (44).
**P10275**	AR	**Androgen receptor (Dihydrotestosterone receptor)**	0.9123	A	Translocation happens after ligand binding and is mediated by filamin, which is thought to disrupt the association between Hsp90 and the receptor in the cytoplasm.
**P84022**	SMAD3	**Mothers against decapentaplegic homolog 3**	0.9123	A	Activated TGF-beta receptor phosphorylates Smad2 and Smad3, which then form a complex with Smad4 and translocate to the nucleus.
**Q13547**	HDAC1	**Histone deacetylase 1**	0.9123	B	In neuroblastoma cells translocation of HDAC1 was reported to the cytoplasm in response to HSV-1 viral infection (45).
**Q15796**	SMAD2	**Mothers against decapentaplegic homolog 2**	0.9123	A	After phosphorylation of receptor-regulated SMADs (SMAD1, SMAD2, SMAD3, SMAD5 and SMAD8) they are recognized by SMAD 4. This complex translocates to the nucleus.
**Q86X55**	CARM1	**Histone-arginine methyltransferase CARM1**	0.9123	B	Nucleus → cytosol translocation mainly occurs during mitosis, but it also occurs out of the cell cycle (46).
**Q9H3D4**	TP63	**Tumor protein 63 (p63)**	0.9123	C	Nuclear localization of p63 was correlated with nuclear accumulation of p53, whereas the presence of nuclear p63 had no apparent effect on patient survival (24–27). The mechanism remains to be elucidated.
**P08238**	HSP90AB1	**Heat shock protein HSP 90-beta**	0.9053	A	Hsp90 has been found in the extracellular region, and also in the nucleus.
**Q13616**	CUL1	**Cullin-1**	0.9034	B	ROC1 promotes CUL1 nuclear accumulation to facilitate its NEDD8 modification (47).
**P10826**	RARB	**Retinoic acid receptor beta**	0.8967	B	This is a nucleocytoplasmic shuttling protein, AFP may inhibit translocation of RAR-beta into the nucleus via competitive binding to RAR-beta with ATRA (48).

**Figure 2.**
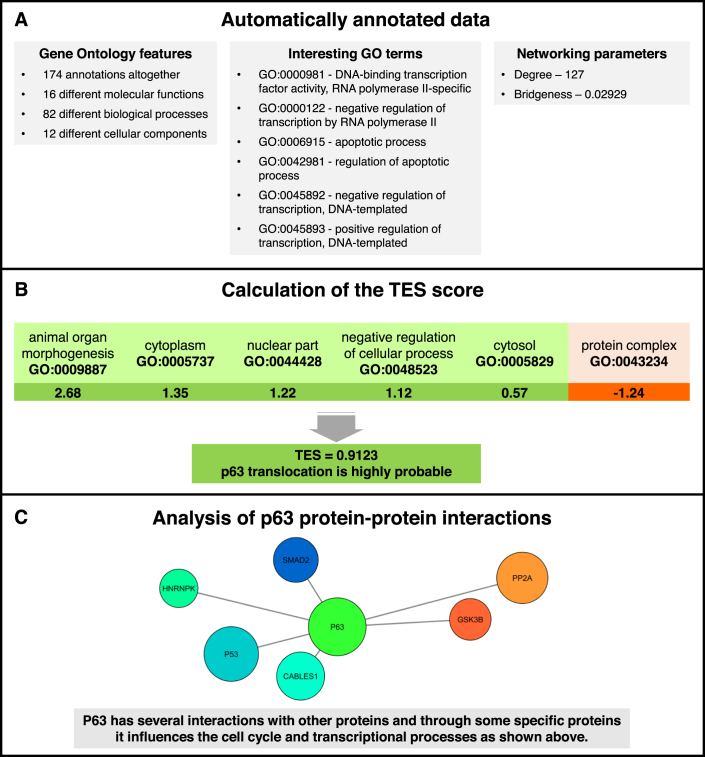
p63, a translocating protein predicted by the XGBoost machine learning algorithm. (**A**) In the left column the Gene Ontology (GO) terms (13,14) that are associated with the p63 protein are summarized, showing that altogether the protein is characterized by 174 annotations. As some of these annotations are redundant, altogether there are 12, 16 and 82 specific GO terms of cellular components, molecular functions and biological processes, respectively. In the right column the degree and the bridgeness value of p63 in the ComPPI database-derived human interactome (3) are shown. In the centre 6 highlighted GO terms show that p63 plays an important role in the regulation of transcription and the apoptotic process. A complete list of associated GO terms was collected by Quick-GO (28) and is available here: https://www.ebi.ac.uk/QuickGO/annotations?geneProductId=Q9H3D4. (**B**) The XGBoost machine learning algorithm (10–12) selected 17 types of features as the best model when calculating the Translocation Evidence Score (TES, see Table 1 and Supplementary Text S6). Out of the 17 features the p63 protein is characterized by 6 GO features and a large degree. For every GO-related feature we have shown the name of the specific GO term and the respective importance value of this GO term. The high TES score shows that the translocation of p63 is highly probable. (**C**) As it is also suggested by some of its major interaction partners shown, the p63 protein is a regulator of transcription and apoptosis. Reviewing the literature, we found that besides the well-known nuclear localization of the p63 protein (24,25) in fact, it also has a validated cytoplasmic localization, too. Moreover, cytoplasmic localization of p63 is a predictor of increased malignancy of some tumours (26,27). This disease-altered localization of p63 is in compliance with our definition for a translocating protein. Thus p63 was correctly predicted by Translocatome as a likely candidate of further experimental studies proving its translocation.

Every protein are shown in the table with their 3 indicators (UniProt ID, Gene name and Protein name) and Translocation Evidence Score (TES) as defined in the main text. The higher the TES score the higher the probability of translocation. Proteins fall into four categories as shown in the fifth column. **(A)** The protein was included in the manually curated 213 translocating protein set. (B) The protein did not appear in our keyword-based searches but was previously shown to be a translocating protein. (C) The protein has not been described as a translocating protein yet, but from the literature we can conclude that its translocation is probable (p63 protein, for more information, see Figure 2). (D) There is no information in the literature about the translocation of this protein (PRKRA). Categories C and D are good candidates for further evaluation. Short summary gives a brief description of the translocation mechanism of each protein having a representative publication cited in categories B and C (for references describing the translocation of proteins in category A see the Translocatome database entry of the respective protein).

The best hit of the XGBoost algorithm, the PTEN protein is a part of the manually curated 213 translocating proteins. As its second best hit, the XGBoost algorithm correctly predicted NF2 (Merlin) as a translocating protein, since NF2 in its dephosphorylated form indeed translocates to the nucleus (23). NF2 is a hub having 48 neighbours and was characterized by 6 out of the 15 Gene Ontology terms that were important according to the best XGBoost model predicting translocation.

Out of the 25 proteins listed on Table 2, the p63 protein (tumor protein 63, TP63) is the only protein, which falls into the category C.) containing ‘proteins having implications in the literature that they are translocating’. p63 is not tagged as translocating in available databases (8,9). p63 is a protein that is physiologically found in the nucleus of human cells (Figure 2). It acts as a transcription factor either activating or repressing specific DNA sequences (24) and it is an essential factor during embryogenesis (25). Besides these conventional functions it is also known that p63 appears in the cytoplasm of adenocarcinoma or prostate carcinoma cells. Moreover, the cytoplasmic localization of p63 results in the increased malignancy of these tumours (26,27). This disease-altered localization of p63 is in compliance with our definition for a translocating protein. Thus, the XGBoost machine learning algorithm correctly predicted the translocation of p63. As p63 is associated with poor survival of cancer patients (26,27) its targeting may serve as a therapeutic option.

With the above examples we demonstrated that the XGBoost machine learning algorithm (10–12) is able to classify previously known proteins effectively and may also predict new translocations correctly. Out of the 25 best hits shown on Table 2 the PRKRA protein (interferon-inducible double-stranded RNA-dependent protein kinase activator A) is the only one, which appears to be a completely new translocating protein candidate. It will be an interesting question of further experimental studies, whether this protein is indeed translocating or shuttling between the cytosol and the nucleus as predicted by the rather equal number of its protein interactions (3) in these two compartments.

### Comparison with similar tools

The existing MoonProt (8) and UniProt (9) databases contain potentially translocating proteins performing multiple biochemical functions or data related to protein translocation, respectively. Out of the 75 human proteins of the latest, 2.0 version of the MoonProt database (accessed on 04/01/2018), 55 proteins were shown in the literature to translocate in a regulated manner (and were included to the Translocatome). The other 20 human moonlighting proteins achieve their multiple functions in the same cellular compartment. Out of the total number of 20 239 human UniProt proteins (accessed on 17 November 2017), we can presume a translocation in 1013 cases based on their UniProt description or subcellular location data. As only 75 (35%) of the 213 Translocatome gold standard proteins were included in the 1013 presumably translocating UniProt proteins, the Translocatome database can greatly supplement this aspect of the UniProt database. From the residual 938 UniProt translocation candidates 25% and 34% were predicted in the Translocatome as high- and low-confidence translocating proteins, respectively. 31% of the 938 UniProt proteins was predicted as non-translocating while 10% of them was not part of the Translocatome database.

## CONCLUSIONS AND FUTURE DIRECTIONS

In summary, Translocatome offers a unique dataset of 213 specifically collected human translocating proteins listing the source of their experimental validation, several details of their translocation mechanism, local compartmentalized interactome as well as their involvement in signalling pathways and disease development. In addition, it provides translocation likelihood values (as Translocation Evidence Scores) for 13 066 human proteins identifying 1133 and 3268 high- and low-confidence translocating proteins, respectively. The assembly of the Translocatome database (Figure 1) combines careful manual curation steps with a state-of-art machine learning prediction protocol. The application examples (Table 2 and Figure 2) show that the Translocation Evidence Score of Translocatome is able to highlight already experimentally verified translocating proteins, which do not evidently appear by simple key word-based search methods, as well as proteins, whose translocation is already very likely from the literature, but has not been directly verified yet. These features position Translocatome as a discovery-tool in the field of protein translocation.

The Translocatome database can be accessed via a user-friendly web-interface providing a quick search function (with UniProt AC autocompletion) and an advanced search to find sets of proteins filtered by their localization, UniProt identifiers, Translocation Evidence Score or Data Complexity Score. The web interface provides eight pre-defined protein sets as download options and a possibility to download the search results. End-user documentation is available as tutorials, detailed descriptions and location-specific tooltips in the HELP menu of the site.

Translocatome is available at http://translocatome.linkgroup.hu. Translocatome is a community-annotation resource, which is helped by its manual curation framework (MCF). MCF allows the users to build in their own experimentally verified translocating proteins. Translocatome will be updated and upgraded annually for minimum 5 years. The Translocatome database is connected to our previously developed, compartmentalized protein–protein interaction database (ComPPI, 3). Thus the improvement of the subcellular localization and interactome data can be easily translated to regular updates of the Translocatome database giving improved protein translocation likelihood values.

We plan to resolve current Translocatome limitations, such as extending the database to other species than humans. Future plans include the extension of positive and negative datasets and localization-based network visualization. Translocating RNAs play a key role in subcellular regulation as well, but their role is even more complex and mysterious. We plan to extend our database and add translocating RNAs, to fill out this gap. The improvement of the data not only means, that Translocatome will have more proteins or more detailed information. In this process the whole database will be updated meaning that the XGBoost machine learning will reappraise the data and provide more even accurate predictions based on the updated data.

In conclusion, the Translocatome database introduced here provides the first dedicated collection of 213 translocating human proteins including their interaction partners in the different subcellular localizations. Importantly, Translocatome gives a Translocation Evidence Score to more than 13 thousand human proteins allowing the assessment of their translocation probability. All these features are accessible in a user-friendly manner. The Translocatome database allows a better comprehension of protein translocation as a systems biology phenomenon, and can be used as a discovery-tool of the field. Since translocating proteins become more and more important therapeutic targets (6,7) Translocatome may contribute to the development of better future therapeutic options.

## DATA AVAILABILITY

The Translocatome database of human translocating proteins can be accessed freely at http://translocatome.linkgroup.hu.

## Supplementary Material

Supplementary Data
