# Supplementary material for: Translocatome: a novel resource for the analysis of protein translocation between cellular organelles
Source: Nucleic Acids Res. 2018 Oct 31;47(Database issue):D495–505. doi: 10.1093/nar/gky1044 (PMC6324082; doi:10.1093/nar/gky1044)
Supplement: Supplementary Data [file gky1044_supplemental_files.pdf]

Supplementary Material to

Translocatome: a novel resource for the analysis of protein translocation between cellular organelles

Péter Mendik[1], Levente Dobronyi[1], Ferenc Hári[1], Csaba Kerepesi[2,3], Leonardo Maia-Moço[1,4], Donát Buszlai[1], Peter Csermely[1] * and Daniel V. Veres[1,5]

[1]Department of Medical Chemistry, Semmelweis University, Budapest, Hungary;
[2]Institute for Computer Science and Control (MTA SZTAKI), Hungarian Academy of Sciences, Budapest, Hungary;
[3]Institute of Mathematics, Eötvös Loránd University, Budapest, Hungary;
[4]Cancer Biology and Epigenetics Group, Research Center of Portuguese Oncology Institute of Porto, Portugal
[5]Turbine Ltd., Budapest, Hungary;
* To whom correspondence should be addressed. Tel: +36-1-459-1500 extension: 60130; Fax: +36-1-266-3802;
Email: csermely.peter@med.semmelweis-univ.hu

Table of Contents

Supplementary Texts ***3***

Supplementary Text S1. Cellular processes not assessed as translocation 3

Supplementary Text S2. Additional considerations in the definition of protein translocation 4

Supplementary Text S3. Manual curation of translocating proteins 5

Supplementary Text S4. Manual curation of non-translocating proteins 7

Supplementary Text S5. Gene Ontology annotation of the proteins 8

Supplementary Text S6. Prediction by the XGBoost machine learning method 9

Supplementary Text S7. Calculation of the Data Complexity Score (DCS) 11

Supplementary Text S8. Calculation of the F1 score 12

Supplementary Text S9. Design and implementation of the Translocatome database 13

Supplementary Tables ***14***

Supplementary Table S1. Number of hits in PubMed and Google Scholar searches 14

Supplementary Table S2. Positive training set 15

Supplementary Table S3. Negative training set 16

Supplementary Table S4. Occurrence of the 11 top high-confidence translocating proteins not part of our Core Data in PubMed searches 17

The Table of Contents is continued on the next page

Table of Contents (continued from the previous page)

Supplementary Figures ... ***18***

Supplementary Figure S1. Workflow of the data acquisition process ... 18

Supplementary Figure S2. Degree and bridgeness of all proteins, as well as the positive and negative training sets ... 19

Supplementary Figure S3. Performance of the widely-used XGBoost machine learning method on the final feature set evaluated by precision-recall and Matthews correlation coefficient curves of hundred 5-fold cross validations ... 20

Supplementary Figure S4. Statistical parameters of the final prediction and translocation evidence-(TES) scores ... 21

Supplementary Figure S5. Data structure of manually curated translocating protein download files 22

Supplementary Figure S6. Data structure of pre-defined download option files ... 24

Supplementary Fig. S7. Data structure of the protein-protein interaction network downloadable file25

Supplementary References ... ***26***

Supplementary Texts

Supplementary Text S1. Cellular processes not assessed as translocation

To increase the focus and clarity of the Translocatome dataset we did not consider co-translational; post-translational delivery-type; cell division-induced; downregulation- or passive diffusion-related phenomena as protein translocation despite of the fact that the subcellular localization of a given protein may change in these situations. Please find a rationale of these considerations in the following paragraphs.

1. **Co-translational delivery.** During the translation process several proteins pass the membrane of the endoplasmic reticulum. We did not consider this phenomenon as protein translocation.
2. **Post-translational delivery.** During post-translational maturation processes several proteins change their localization in the cell, e.g. move from the endoplasmic reticulum to the Golgi apparatus where they may undergo additional post-translational modifications. We did not consider these protein movements as translocation as they are part of the post-translational maturation process and they are mostly not induced by a specific signal. We considered protein translocation as a phenomenon which happens after the protein reached its final destination and started to function.
3. **Protein translocation during the cell division process.** During mitosis or meiosis the subcellular membrane structure becomes reorganized. As one of the primary changes the integrity of the nuclear membrane is massively decreased, thus in these parts of the cell cycle nucleocytoplasmic translocation cannot be observed as in G0 phase. Therefore, protein translocations which were described during the cell division process were regarded with criticism and were added to the Translocatome database only if they had a functional meaning and not merely happened as a consequence of the cell division process.
4. **Protein movements related to downregulation.** Downregulation of several proteins (e.g. that of plasma membrane receptors) is achieved by their internalization to endocytotic vesicles and finally to the lysosome. We did not consider these phenomena as protein translocation.
5. **Protein movements related to passive diffusion.** If a protein is small enough it can passively transit through some subcellular membranes (where the best known example is the nuclear pore). This phenomenon results in a multicompartmental localization of these proteins. We, therefore, regarded the translocation of small-size proteins with special criticism.

Supplementary Text S2. Additional considerations in the definition of protein translocation

1. **Definition of subcellular compartments.** We considered six subcellular compartments: cytoplasm, extracellular space, mitochondria, nucleus, membrane, secretory-pathway based on the compartmentalized protein-protein interaction database, ComPPI (http://comppi.linkgroup.hu, 1). We used these six major localizations because there is enough, comprehensive, high confidence information to create a localization-specific interactome (localization information is usually a result of a high-throughput method, therefore using more detailed localization items could increase the level of noise and create more bias in the data used). There are lower level functional organelles in cells (e.g.: proteasome, lysosome etc.), but their interactomes are not always well characterized and are usually not separated in system-level studies.

2. **Intra-compartmental translocations.** Most current methods thus do not make it possible to evaluate translocations happening inside major localizations. Thus, these “intra-compartmental” translocations where not included to the Translocatome database. However, we always saved the minor localization of translocating proteins, thus when systematic studies will provide a better resolution then the Translocatome database can be updated to cover these intra-compartmental translocations as well.

3. **Intra-compartmental moonlighting proteins.** Moonlighting proteins are known as proteins that are capable of executing different biochemical processes and are collected in the MoonProt 2.0 database (http://moonlightingproteins.org, 2). We considered moonlighting proteins as translocating proteins if their moonlighting involved more than a single subcellular organelle. There are proteins that have different function inside one subcellular organelle. As a specific example of the intra-compartmental translocation mentioned in point 2, these intra-compartmental moonlighting proteins were not included to our database.

4. **Extent of translocation.** We did not consider translocation as an 'all-or-none' phenomenon. Thus we included proteins as translocating proteins when the ratio of a protein in two given compartments changed substantially (e.g.: a protein that was mainly localized to the cytoplasm became mainly localized to the nucleus, however, this change was not complete, and there is still a small amount of the protein, which can be found in the cytoplasm).

5. **Participation in translocation.** Those papers, where "protein A" mediated the translocation of "protein B", or "protein A" interacted with the translocating "protein B" were considered only from the point of "protein B" and not regarding "protein A".

6. **Definition of regulated movement of a protein.** We collected translocating proteins participating in different cellular signalling mechanisms and responses, where the regulated movement of the protein was achieved by a signal. In addition, we considered regulated movement of the protein when the differing localization was achieved by the onset of a pathological condition (like e.g. cancer).

Supplementary Text S3. Manual curation of translocating proteins

The manual curation team consisted of biochemists, bioinformaticians, cell biologists, molecular biologists and physicians. To ensure high quality manual curation every curated entry was assessed in the following way. After a paper was carefully reviewed by the primary curator it was subsequently reviewed by a senior member of the Translocatome team. If the data contained any inconsistencies or wasn't straightforwardly understandable in any way a discussion was initiated (involving at least one other member of the team) and the problematic information became corrected. The entry became accepted to the database only after every topic in question has been adequately cleared and the entry has been cross-checked by at least 3 independent experts in the process.

We give a flow chart of the manual curation process on Supplementary Figure S1. Our method of the curation process was similar to the methodology of previously published, manually curated databases like the MoonProt database (2).To find relevant scientific papers for building the gold standard positive dataset of 213 translocating proteins we used the PubMed and Google Scholar search engines. Key word-based searches resulted in a high number of hits as shown in Supplementary Table S1. After the initial reading process we realized that protein translocation has no unified definition and the word "*translocation*" is also used in a genomic sense to mark translocation of DNA segments or referring to the translocation of RNA on the ribosome. We have summarized our considerations for the definition of protein translocation in Supplementary Texts 1 and 2. This led to key word combinations of *protein translocation, nucleocytoplasmic translocation, nuclear translocation, cytoplasmic translocation and mitochondrial translocation*. We also realized that protein translocation is often referred as "*shuttling*" so we used this keyword, too. As shown on Table S1 these, more adequate key words and key word combinations significantly reduced the number of hits.

The manual curation process started on 25th October 2015 and finished at the end of 2017. During the browsing/filtering/selection process of the refined key word based searches we applied the following preferences and techniques:

- recent papers were preferentially checked, especially regarding review papers;
- several experimental papers were "traced back" using the relevant references of review papers and databases;
- papers appearing as "best match sorting" of PubMed searches and starting hits of Google Scholar searches were preferentially checked;
- key word searches were refined using word combinations in quotation marks;
- key word searches were refined using restrictions to "title" or "title/abstract";
- searches were considering only human proteins;
- only peer-reviewed papers were considered;
- papers on chromosomal, RNA or other types of translocations not related to protein translocation were not considered;
- the exclusion criteria summarized in Supplementary Text S1 were applied;
- the additional exclusion criteria summarized in Supplementary Text S2 (such as intra-compartmental translocation, intra-compartmental moonlighting, the necessity to have a regulated translocation movement, etc.) were applied;
- papers describing redundant information on previous hits were not considered.

As a result of this filtering/selection process we read over a hundred review papers and over a thousand pre-selected experimental papers submitting them to the manual curation process described in the starting paragraph of this Supplementary Text. Since we did not record the number of irrelevant articles from the start, we may only give estimates of the total papers read here. In addition, we also used direct, name-based searches for well-known translocating proteins, like nuclear hormone receptors, p53, EGFR, HIF1-alpha and other proteins, mentioned in review papers or in databases such as ComPPI (1) or MoonProt (2).

During the detailed reading process of the selected papers we first cross-checked all exclusion criteria mentioned in the screening process (including those listed in Supplementary Texts S1 and S2). We included only those proteins to our Core Data, where the papers were discussing not only the mere fact of their translocation but also the details of their translocation mechanism (e.g. its regulation or mechanism related to the structure of the translocating protein) based on experimental validation. However, these details of the translocation process were not discussed in equal depths in different articles. To give an information on the richness of data behind each of the 213 entries of our database we created the Data Complexity Score as described in the main text and in Supplementary Text S7.

We finished data collection when an increasing amount of former hits appeared in our new searches completing our Core Data having 213 human translocating proteins described in the 238 papers available at the link in the footnote[1].This does not mean that the number of 213 manually curated translocating proteins of this Core Data (see here http://translocatome.linkgroup.hu/coredata) would give a complete list of human translocating proteins, as it is shown by Table 1 of the main text and Supplementary Table S4, where 11 out of the best 25 predictions turned to be already known translocating proteins not being part of our Core Data. This is one of the reasons why we created the Manual Curation Framework (MCF) to allow the continuous possibility to add further entries to the manually curated positive dataset. MCF entry suggestions will be reviewed with the same rigor described for the initial manual curation process. In addition, we complemented the manually curated information on translocating proteins with the efficient machine learning tool, XGBoost (3-5) to predict the translocation likelihood of approximately 13 000 human proteins as described in the main text and in Supplementary text S6 in detail.

---

[1] https://www.ncbi.nlm.nih.gov/pubmed?term=24491427+24434356+23435424+24074954+15071501+21900948+19210988+24113167+26449824+26079448+26520802+23955340+25989275+26406376+26056081+26602019+25684142+25823029+26648570+27013058+17308100+24667139+24695740+16772870+26942675+24725411+26516353+26387538+26549027+26470026+26499805+27292796+16390708+19524307+26827288+19808100+24735540+19718473+26969532+21419860+18760948+19854831+22583914+26181205+26379505+26643147+10749215+18974300+25589722+23814078+17123511+11818509+24954011+11679632+27641332+9765220+17786044+20498072+18075313+26559910+27572958+21469768+9822602+9039962+8643491+22345668+12628186+11517310+22534175+16640563+23707396+11231586+16162498+11756542+1985107+22684108+12649597+24841202+23333404+24013206+10477286+15809060+20132536+10512882+27099442+25609610+27346674+27913144+1532584+26891695+24573109+15485931+11301021+14505568+22639060+11043577+28149448+7925301+24324740+9672244+11705999+7782294+15084609+10187816+22186421+24628430+7748180+14733946+12432920+27496138+18636433+18716285+25835495+19815064+27875245+9636171+20826166+22482906+10397761+10811825+20674093+17516504+15496412+1312324+18204201+16782877+22932683+18515545+10973496+11559828+27428427+19216841+19160485+18045535+26319354+25862818+18337556+19651895+10744629+22832227+19503814+18238777+9679058+17289031+27245214+17157415+22178385+26462148+17535848+25635431+16110492+28235034+12115727+20346347+20308327+16807357+28515276+10547363+21157379+27721408+20511593+16403913+10508860+28552616+28504714+12011459+25893308+20651736+24196791+25961505+18334557+22885005+22824301+22391300+16215984+26738429+22692200+27251589+25701194+26500058+26358502+26633708+25882840+24965109+27272778+24784232+24489995+28811933+25893289+25164084+22473997+27462018+25755279+24959884+27941888+26900797+27510036+16375604+23128389+14715249+17595320+22623727+17967441+26651356+16792529+15314173+18973764+12015613+28812328+15728466+23979357+12191473+19114992+23384547+18061509+14769937+14652813+22296597+11925436+28863181+17218261+22989880+20148342+15951807+15803152+25926267+17003494+12809600+15623571+9660801+20605787+16818237+20937816+16951195+10811646+17560175+25299576+17645779%5Buid%5D&cmd=DetailsSearch&log$=activity

Supplementary Text S4. Manual curation of non-translocating proteins

The negative dataset could not be searched using specific keywords as we did in the case of the gold standard positive translocating set, since there are no keywords to identify those proteins that are not translocating. Thus, we designed a different approach using the following considerations.

1. **Database filtering to reveal proteins having only a single minor localization.** Our first approach was to find proteins that only have only a single minor localization, which makes it rather unlikely that they translocate. In order to filter these proteins, we assessed the data of the compartmentalized protein-protein interaction (ComPPI, 1) the UniProt (6) and the Human Protein Atlas (HPA, 7) databases. We found 844 single minor localization proteins in ComPPI. From these proteins 98 had a single localization in UniProt. From these 98 entries 60 cases had the same localization in both databases. From these 60 proteins 18 had localization information in the HPA database. From these 18 proteins 11 proteins had the same localization in all 3 databases. These 11 proteins having the same localization in all 3 databases were included to the negative dataset. To extend this dataset we decided to include those proteins, which had the same, single minor localization in ComPPI (1) and HPA (7). To achieve this we started the filtration process with HPA. Here we found 1001 proteins which had only a single validated localization. We could map 996 SwissProt UniProt IDs to these proteins. We found 995 of these proteins in ComPPI, out of which 18 proteins were found, which had only a single minor localization in the complex dataset of ComPPI having close to 200 thousand localizations for human proteins. 7 of these proteins did not have localization data in UniProt (6). Thus, altogether this process resulted in 18 proteins that have only a single subcellular localization.
2. **Proteins with experimentally validated mono-compartment localization.** 29 proteins total, e.g. ASAT or GPT1. Note that among these proteins may have multi-compartmental localization achieved by high-throughput methods. Such proteins were not included to the search results described in point 1. Here we did include them, since we considered experimental validation more convincing than high-throughput results.
3. **Proteins with diffuse multicompartment localization.** Example of the 4 proteins with diffuse multicompartment localization: PEX12.
4. **Proteins anchored to the cytoskeleton.** Examples of the 16 proteins anchored to the cytoskeleton: titin or myosin.
5. **Docked proteins to the DNA or to the membrane.** Examples of the 26 DNA-bound proteins TAF1B or GATA3. Examples of the 64 membrane-bound proteins: Na/K ATPase or RYR.

Altogether these considerations resulted in 139 non-translocating proteins of the negative dataset (the 18 proteins of the search described in point 1 also appeared in later searches showing the integrity of the approach).

Supplementary Text S5. Gene Ontology annotation of the proteins

For each ComPPI protein (including the training set) we extracted the associated GO terms (8,9). However, in some cases a UniProt (6) entry is associated with a given GO term but not all of the ancestors of this GO term. As an example, the Merlin (P35240) protein entry is associated with the GO term “negative regulation of cell proliferation” (GO:0008285) but not its ancestor GO terms “regulation of cell proliferation” (GO:0042127), “regulation of cellular process” (GO:0050794), “regulation of biological process” (GO:0050789), “biological regulation” (GO:0065007) and “biological process” (GO:0008150)." We solved this problem in the same way as described in earlier studies (5,10). We downloaded the basic version of the Gene Ontology database (with the database filename “go-basic.obo”) and by walking upward in the GO hierarchy, we added all of the ancestor GO terms to the proteins. Note that “go-basic.obo” is guaranteed to have a hierarchical organization where annotations can be followed in the structure of the term-hierarchy. The final feature table contains 13 066 proteins and 21 020 binary (true/false) GO features.

Supplementary Text S6. Prediction by the XGBoost machine learning method

Labels of the binary classification. The target variable (labels) of the binary classification has value 1 for the 160 manually curated translocating proteins ("translocating class") and value 0 for the 139 non-translocating proteins (non-translocating class).

Feature selection on the training set. The well-established and widely used XGBoost machine learning algorithm (3-5) is capable of selecting the most important features by building small decision trees of the most important features and gradually refining the models by adding new trees. We started the feature selection process using the set of 21 020 annotated GO features for the whole training set (n=299). We evaluated the XGBoost-selected feature sets by 5-fold cross-validation (in which we split the data into 5 random parts and in each round, used 4 parts to train and evaluated the prediction on the fifth part) and measured the area under the curve of the receiver operating characteristic curve (AUC,11). For every feature set, we repeated this process 100 times and computed the average AUC.

Final prediction for all the 13 066 proteins. This final feature set and the final model parameters was used to predict translocation for all the 13 066 proteins in the database. The selected features of the model with the best ROC AUC value is shown on Table 1 of the main text with their importance values calculated from the leaf-scores of the one-depth trees of the final XGBoost model (https://github.com/kerepesi/translocatome_ml/blob/master/Results/GO_features.csv-imp-n_est80-thr0.02-table.csv-add_degree_bridgeness.csv_Trees-n_est80-max_d1.txt). Using this feature set we calculated the Translocation Evidence Score characterizing the translocation probability of each of the 13 066 proteins in the database as described in a the main text.

Evaluation measures for binary classification. TP (true positive) is the number of positives that are predicted as positives. TN (true negative) is the number of negatives that are predicted as negatives. FP (false positive) is the number of negatives that are predicted as positives. FN (false negative) is the number of positives that are predicted as negatives. In our context "positive" means "translocating", "negative" means "non-translocation". Precision, recall (or true positive rate), fall-out (or false positive rate), F1 score and Matthews correlation coefficient were computed by Equations 1 to 5, respectively:

$$precision := \begin{cases} \frac{TP}{TP + FP}, & \text{if } TP + FP \neq 0, \\ 1, & \text{otherwise.} \end{cases} \quad (1)$$

$$recall := \frac{TP}{TP + FN}, \quad (2)$$

$$fall - out := \frac{FP}{TN + FP} \quad (3)$$

$$F1\ score := \begin{cases} \frac{2 \cdot precision \cdot recall}{precision + recall}, & \text{if } precision + recall \neq 0, \\ 0, & \text{otherwise.} \end{cases} \quad (4)$$

$$MCC := \begin{cases} \dfrac{TP \cdot TN - FP \cdot FN}{\sqrt{(TP + FP)(TP + FN)(TN + FP)(TN + FN)}}, & \text{if}\,(TP + FP)(TN + FN) \neq 0, \\ 0, & \text{otherwise.} \end{cases} \quad (5)$$

To evaluate the final prediction of the XGBoost method (3-5), we plotted the receiver operating characteristic curve (ROC, Supplementary Figure S4A). The performance of the model was defined as the area under the curve of the receiver operating characteristic curve (AUC, 11). The receiver operating characteristic curve (ROC) is defined by the point pairs of recall (or true positive rates)and fall-out (or false positive rates) at different threshold settings (11). We show the ROC curves, as well as precision-recall curves and Matthews correlation coefficient curves of 100 five-fold cross-validation runs on Figure 1C of the main text and Supplementary Figure S3, respectively, all using the final feature set (see Table 1 of the main text). All showed a high performance as discussed in the main text and legend related to Figure 1C and the legend of Supplementary Figure S3.

Supplementary Text S7. Calculation of the Data Complexity Score (DCS)

The data complexity score (DCS) characterizes the information content of the manually curated protein-associated data of the Translocatome database. DCS is calculated as a weighted measure. The following information can increase the DCS:

Information category	Weight of the respective information
**well described translocation mechanism**	3
**protein structural background of the translocation**	3
**known biological processes in the subcellular compartments**	2
**known interactions in the subcellular compartments**	1
**affected signalling pathway is discovered**	1
**known pathological function of the translocating protein**	1
**type of the pathological condition affected by translocation**	1
**exact pathology for which the translocation is an underlying factor is discovered**	1
**detection method of the cellular localizations is known**	1

After summing the weight values that are true for a given entry we divide this number with the maximal achievable DCS score of 23. Thus, DCS is a weighted and normalized average of the information categories, which can vary between the minimum of zero and the maximum of 1.

Supplementary Text S8. Calculation of the F1 score

Contribution of the various GO-related and network-related features selected by the XGBoost machine learning algorithm (3-5) made us possible to define the Translocation Evidence Score (representing the likelihood of the translocation of a given protein) for all the 13 066 human proteins of Translocatome as described in the main text. The Translocation Evidence Score gave the possibility to define a cut-off value, below which proteins were considered as non-translocating. To define this cut-off value, we used the parameter, F1 score (also called as F-measure, see Equation 4 in Supplementary Text S6, and supplementary reference 12). To calculate the F1 score we defined true positive hits as the positive training set proteins that has been predicted as translocating, true negative hits as the negative training set proteins that has been predicted as negative, false positive hits as the negative training set proteins that has been predicted as translocating and false negative hits as the positive training set proteins that has been predicted as non-translocating proteins – as described in Supplementary Text S6 in more detail.

Supplementary Text S9. Design and implementation of the Translocatome database.

The Translocatome database has altogether 13 066 human protein entries. The core dataset is the 213 manually curated human translocating proteins. Our primary aim was to implement this database as a user-friendly application which is easily browsable, understandable and has powerful search and download options. The website is designed to satisfy the needs of different disciplines providing data addressing several aspects of the translocation phenomenon.

The database has an industry standard-level software design by a multidisciplinary team (database expert, informatician, bioinformatician, physician, graphic expert). The Translocatome website is designed as a client-server architecture: a NodeJS back-end serves the requests and fetches data from the MongoDB database, while the front-end, implemented with React.js displays this data in a user-friendly way.

We selected third-party tools and technologies that favour scientific reproducibility and open accessibility, including the Ubuntu Linux 16.04 operating system (http://ubuntu.com), the nginx HTTP server (http://nginx.org), the MongoDB 3.2 database server (https://www.mongodb.com), the git version control system (http://git-scm.com), the NodeJS 6.10 (https://nodejs.org/en) Javascript server runtime with the Express.js 4.15 framework (http://expressjs.com) and the React.js 15.4 (https://reactjs.org) JavaScript framework.

The dataset can be browsed and searched by various user preferences. Protein names are auto-completed using UniProt (6) accession numbers. The advanced search option gives the choice of translocation direction (from one selected cellular compartment to another), as well as the range of Translocation Evidence Score and Data Complexity Score. During searching a NodeJS script automatically generates a downloadable version of the current data. Furthermore, we provide pre-defined download sets.

The files forming the base of the Translocatome database can be generated with a simple SQL query from our previously developed, MySQL-based compartmentalized protein-protein interaction database (ComPPI, 1). This feature connects the two databases. Thus the improvement of the subcellular localization and interactome data can be easily translated to regular updates of the Translocatome database giving improved protein translocation probability values.

Supplementary Tables

Supplementary Table S1. Number of hits in PubMed and Google Scholar searches

**Key words**	**Unrestricted search** PubMed/Google Scholar*	**Reviews only** available in PubMed	**Filtered to human** available in PubMed
translocation	297 319/133 000 *(329 989/154 000)***	32 602 *(36 096)*	144 728 *(160 749)*
protein translocation	251 037/99 000 *(278 900/109 000)***	26 922 *(29 802)*	116 726 *(130 928)*
shuttling	3420/21 000 *(4001/23 000)***	402 *(466)*	1743 *(1972)*
nucleocytoplasmic translocation	2711/4000 *(3016/8000)***	347 *(403)*	1551 *(1740)*
nuclear translocation	43 891/64 000 *(50 023/72 000)***	3568 *(3955)*	25 130 *(28506)*
cytoplasmic translocation	84 923/35 000 *(93 122/45 000)***	8902 *(9799)*	38 047 *(42 454)*
mitochondrial translocation	25 239/24 000 *(28 653/48 000)***	2908 *(3318)*	10 409 *(12 135)*
**Total**	**299 216/133 000** ***(332 304/154 000)*****	**32 858** ***(36 398)***	**145 528** ***(161 674)***

To find relevant scientific papers for building the gold standard positive dataset of 213 translocating proteins we used the PubMed and Google Scholar search engines searching for the keywords: *translocation, protein translocation, shuttling, nucleocytoplasmic translocation, nuclear translocation, cytoplasmic translocation and mitochondrial translocation.* Articles found were manually curated using the definition and the exclusion criteria as discussed in Supplementary Text S3. This table shows the number of PubMed and Google Scholar search results for each keyword separated by a slash. In the first column the results of the unrestricted search are shown. We also included the number of search results if we filtered for only review articles or only human studies. The manual curation process started on the 25.09.2015. and the numbers show the results restricted until that time. Italic numbers in parentheses refer to the number of papers restricted until the end of the manual curation process which was on 31 December 2017. For the assembly of the data in this table both databases were accessed on 4th October 2018.

*Google Scholar hits were rounded to thousands. The total of Google Scholar counts refers to the search of "translocation" since the addition of "OR shuttling" term did not improve the number of hits. Without any restrictions Google Scholar gives over 2 million hits for "translocation".

**Italic numbers in parentheses refer to the number of papers restricted until the end of the manual curation process which was on 31 December 2017.

Supplementary Table S2. Positive training set

UniProt AC	Gene name	UniProt AC	Gene name	UniProt AC	Gene name	UniProt AC	Gene name
**Q8IZP0**	ABI1	Q9UER7	DAXX	P17936	IGFBP3	P12272	PTHLH
**P00519**	ABL1	Q08211	DHX9	P24593	IGFBP5	P49023	PXN
**P42684**	ABL2	Q13316	DMP1	P24592	IGFBP6	P20339	RAB5A
**Q9NR19**	ACSS2	P26358	DNMT1	Q92985	IRF7	P63000	RAC1
**P35869**	AHR	Q01094	E2F1	Q92830	KAT2A	Q06609	RAD51
**Q9GZX7**	AICDA	Q15029	EFTUD2	Q92831	KAT2B	P10276	RARA
**P55008**	AIF1	P00533	EGFR	P01116	KRAS	Q08999	RBL2
**O95831**	AIFM1	P18146	EGR1	Q9UN81	L1RE1	P18754	RCC1
**P31749**	AKT1	Q9Y6B2	EID1	Q14847	LASP1	O94761	RECQL4
**P09917**	ALOX5	P06730	EIF4E	P02545	LMNA	Q04206	RELA
**Q8NAG6**	ANKLE1	Q15717	ELAVL1	Q02750	MAP2K1	P51449	RORC
**Q7Z6G8**	ANKS1B	P78545	ELF3	P28482	MAPK1	P62829	RPL23
**P10275**	AR	P06733	ENO1	P27361	MAPK3	P23396	RPS3
**P10398**	ARAF	Q15303	ERBB4	P29966	MARCKS	Q13950	RUNX2
**P15336**	ATF2	P03372	ESR1	O00255	MEN1	Q01105	SET
**P18848**	ATF4	P09038	FGF2	P15941	MUC1	O00141	SGK1
**P35670**	ATP7B	Q12778	FOXO1	P19878	NCF2	Q96EB6	SIRT1
**P54252**	ATXN3	O43524	FOXO3	P19338	NCL	P14672	SLC2A4
**P15291**	B4GALT1	P04406	GAPDH	O95644	NFATC1	Q15797	SMAD1
**Q16611**	BAK1	P10071	GLI3	Q14934	NFATC4	P48431	SOX2
**Q07812**	BAX	O75496	GMNN	Q16236	NFE2L2	P48436	SOX9
**Q07817**	BCL2L1	P04899	GNAI2	P19838	NFKB1	Q9NYA1	SPHK1
**O15392**	BIRC5	P56524	HDAC4	Q00653	NFKB2	Q12772	SREBF2
**P38398**	BRCA1	Q9UQL6	HDAC5	P29474	NOS3	Q07955	SRSF1
**P27797**	CALR	Q16665	HIF1A	P04150	NR3C1	P42224	STAT1
**O14936**	CASK	P16403	HIST1H1C	Q6X4W1	NSMF	P40763	STAT3
**P14635**	CCNB1	P52789	HK2	Q8TAK6	OLIG1	Q13043	STK4
**Q16589**	CCNG2	P09429	HMGB1	P49585	PCYT1A	P32856	STX2
**P50750**	CDK9	P09651	HNRNPA1	O15534	PER1	Q15561	TEAD4
**P46527**	CDKN1B	O14979	HNRNPDL	P00558	PGK1	O14746	TERT
**P49918**	CDKN1C	P52597	HNRNPF	P14618	PKM	P36897	TGFBR1
**P17676**	CEBPB	P31943	HNRNPH1	P00749	PLAU	P21980	TGM2
**O14757**	CHEK1	P61978	HNRNPK	P37231	PPARG	P10828	THRB
**Q99828**	CIB1	Q00839	HNRNPU	Q00005	PPP2R2B	Q9BSI4	TINF2
**P49759**	CLK1	P01112	HRAS	Q15172	PPP2R5A	P62328	TMSB4X
**Q16526**	CRY1	P07900	HSP90AA1	Q14738	PPP2R5D	P04637	TP53
**P56545**	CTBP2	P08238	HSP90AB1	P30041	PRDX6	Q13114	TRAF3
**P35222**	CTNNB1	P11142	HSPA8	P17612	PRKACA	Q9NSU2	TREX1
**Q14247**	CTTN	Q02363	ID2	P78527	PRKDC	P68543	UBXN2A
**P99999**	CYCS	P18065	IGFBP2	P60484	PTEN	P46937	YAP1

Supplementary Table S2 contains the 160 manually curated translocating proteins that translocate under physiological conditions and thus were selected as elements of the positive training set.

Supplementary Table S3. Negative training set

UniProt AC	Gene name	UniProt AC	Gene name	UniProt AC	Gene name	UniProt AC	Gene name
**A6NGB9**	WIPF3	P08922	ROS1	P48764	SLC9A3	Q92824	PCSK5
**A8MQ14**	ZNF850	P0C024	NUDT7	P49790	NUP153	Q92908	GATA6
**A8MUV8**	ZNF727	P0CJ78	ZNF865	P51946	CCNH	Q96HA9	PEX11G
**B4DU55**	ZNF879	P10635	CYP2D6	P54277	PMS1	Q96LT4	SAMD8
**C9JN71**	ZNF878	P10721	KIT	P60709	ACTB	Q96MW1	CCDC43
**E7ETH6**	ZNF587B	P11055	MYH3	P62068	USP46	Q99595	TIMM17A
**O00391**	QSOX1	P12883	MYH7	P68133	ACTA1	Q99705	MCHR1
**O00623**	PEX12	P13533	MYH6	Q12824	SMARCB1	Q99965	ADAM2
**O14576**	DYNC1I1	P13535	MYH8	Q12931	TRAP1	Q9BRQ3	NUDT22
**O14925**	TIMM23	P13569	CFTR	Q13423	NNT	Q9BRT6	LLPH
**O14964**	HGS	P14416	DRD2	Q14108	SCARB2	Q9BWM7	SFXN3
**O14975**	SLC27A2	P16278	GLB1	Q14249	ENDOG	Q9HCE1	MOV10
**O14983**	ATP2A1	P16473	TSHR	Q15388	TOMM20	Q9NRP2	CMC2
**O60341**	KDM1A	P16581	SELE	Q15722	LTB4R	Q9NS69	TOMM22
**O60563**	CCNT1	P17174	GOT1	Q16678	CYP1B1	Q9NUJ7	PLCXD1
**O75027**	ABCB7	P18847	ATF3	Q16822	PCK2	Q9NZ42	PSENEN
**O75192**	PEX11A	P18859	ATP5J	Q16878	CDO1	Q9NZ52	GGA3
**O75694**	NUP155	P19367	HK1	Q53T94	TAF1B	Q9NYW8	RBAK
**O94822**	LTN1	P19404	NDUFV2	Q71U36	TUBA1A	Q9P0Z9	PIPOX
**O94826**	TOMM70	P20309	CHRM3	Q7Z406	MYH14	Q9UH99	SUN2
**O94901**	SUN1	P20585	MSH3	Q7Z412	PEX26	Q9UHC1	MLH3
**O95182**	NDUFA7	P21439	ABCB4	Q86Y39	NDUFA11	Q9UHD2	TBK1
**O95198**	KLHL2	P21817	RYR1	Q86YV0	RASAL3	Q9UJ83	HACL1
**O96008**	TOMM40	P22303	ACHE	Q8IWY9	CDAN1	Q9UJM8	HAO1
**P01106**	MYC	P23771	GATA3	Q8IXM3	MRPL41	Q9UKU7	ACAD8
**P03923**	MT-ND6	P24298	GPT	Q8N9L9	ACOT4	Q9UKX2	MYH2
**P04629**	NTRK1	P24752	ACAT1	Q8N9W6	BOLL	Q9UL17	TBX21
**P05023**	ATP1A1	P35548	MSX2	Q8NGJ1	OR4D6	Q9UQ90	SPG7
**P05026**	ATP1B1	P35558	PCK1	Q8TD30	GPT2	Q9Y259	CHKB
**P05412**	JUN	P35579	MYH9	Q8TES7	FBF1	Q9Y2Q9	MRPS28
**P05496**	ATP5G1	P35580	MYH10	Q8WVN6	SECTM1	Q9Y2W1	THRAP3
**P06276**	BCHE	P35749	MYH11	Q8WVX9	FAR1	Q9Y3A0	COQ4
**P06400**	RB1	P36542	ATP5C1	Q8WXD0	RXFP2	Q9Y584	TIMM22
**P07949**	RET	P40939	HADHA	Q8WZ42	TTN	Q9Y5J7	TIMM9
**P08684**	CYP3A4	P48058	GRIA4	Q92621	NUP205		

Supplementary Table S3 contains the 139 manually collected non-translocating proteins that were collected based on the considerations discussed in Supplementary Text S4 and form the negative training set.

Supplementary Table S4. Occurrence of the 11 top high-confidence translocating proteins not part of our Core Data in PubMed searches

Protein name	UniProt ID	PubMed ID	PubMed search term						
			"translocation"	"protein translocation"	"shuttling"	"nucleo-cytoplasmic translocation"	"nuclear translocation"	"cytoplasmic translocation"	"mitochondrial translocation"
NF2	P35240	24726726<br>20178741	**21**<br>29709009	**0**	**1**	**0**	**4**<br>29709009	**1**	**0**
TULP3	O75386	11375483	**3**<br>11375483	**0**	**0**	**0**	**0**	**0**	**0**
SNCA	P37840	18440504*	**5**	**0**	**2**	**0**	**0**	**0**	**0**
FGFR2	P21802	15654655	**32**<br>16365892<br>15654655	**0**	**0**	**0**	**5**<br>16365892<br>15654655	**0**	**0**
MTOR	P42345	11114166*	**14**<br>29512299<br>28694500<br>21822208	**0**	**2**<br>26097872	**0**	**3**<br>21822208	**0**	**0**
GSK3B	P49841	17438332*	**7**	**0**	**0**	**0**	**0**	**0**	**0**
EIF6	P56537	21084295	**2**<br>21084295	**0**	**1**<br>21084295	**0**	**0**	**0**	**0**
HDAC1	Q13547	15897453<br>20037577<br>27669993	**16**<br>24658119	**1**	**2**	**0**	**9**<br>24658119	**0**	**0**
CARM1	Q86X55	19843527<br>17848568<br>19208762	**4**<br>29681515<br>19843527	**0**	**1**	**0**	**2**<br>29681515	**0**	**0**
CUL1	Q13616	26068074**<br>11027288	**13**<br>21247897	**0**	**0**	**0**	**4**<br>21247897	**1**	**0**
RARB	P10826	19501957*	**5**	**0**	**0**	**0**	**0**	**0**	**0**

Supplementary Table S4 contains the number of papers **in boldface** retrieved from Title/Abstract (in case of MTOR and HDAC1 Title/Title and HDAC1/Title, respectively) restricted PubMed searches. Nine digit PubMed IDs refer to those papers among these, where experimental evidence of translocation was listed. Experimental evidence for the translocation of three proteins (SNCA, GSK3B and RARB) were not found in keyword searches of their UniProt names, since these proteins were mentioned by different name versions in the papers describing the experimental evidence for their translocation.
*The name of the protein was not the same as its UniProt name in the paper.
**Note that this paper refers to an "SCF Ubiquitin Ligase Complex" which includes CUL1.

Supplementary Figures

Supplementary Figure S1. Workflow of the data acquisition process

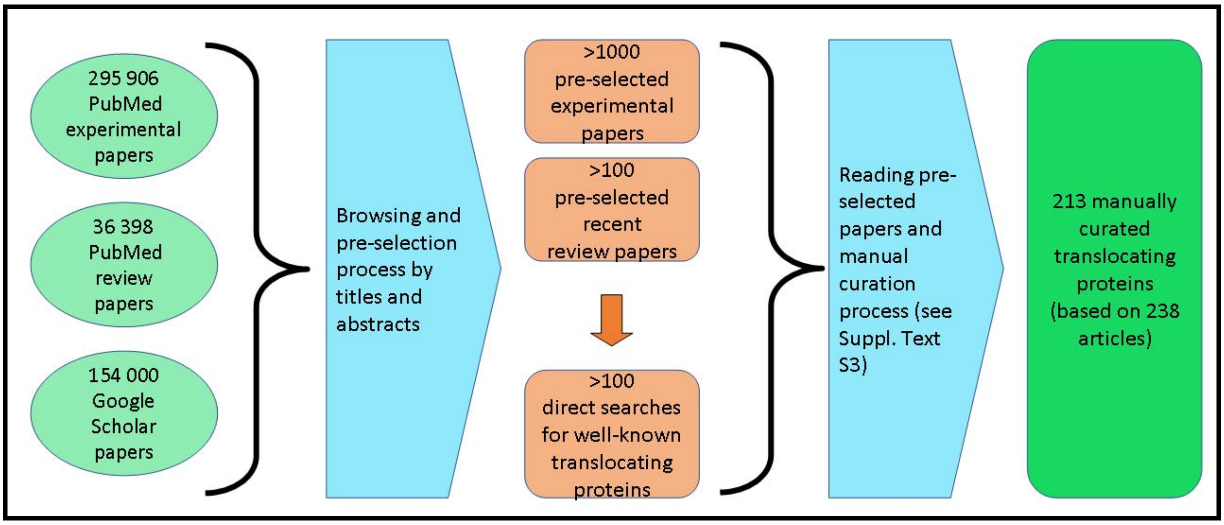

The figure shows the flow-chart of our manual curation process, which started on 25th October 2015 and finished at the end of 2017. The curation process was similar to the methodology of previously published, manually curated databases like the MoonProt database (2). We started from a pool of 295, 36 and 154 thousand PubMed experimental papers, PubMed reviews and Google Scholar papers, respectively, as shown on Supplementary Table S1 in detail. We especially considered recent review papers as references to more detailed searches and studies. The browsing/pre-selection process had the preferences described in Supplementary Text S3 in detail. As a result of this process we read more than a thousand research papers and over hundred reviews. We also made over a hundred direct searches for translocating proteins suggested by reviews and databases like ComPPI (1) or MoonProt (2). Applying the selection criteria of Supplementary Texts 1 through 3 during the careful manual curation process described in the starting paragraph of Supplementary Text S3, we finally selected the 238 papers which describe experimental evidence of the translocation of 213 human proteins forming the Core Data of our database (see here: http://translocatome.linkgroup.hu/coredata).

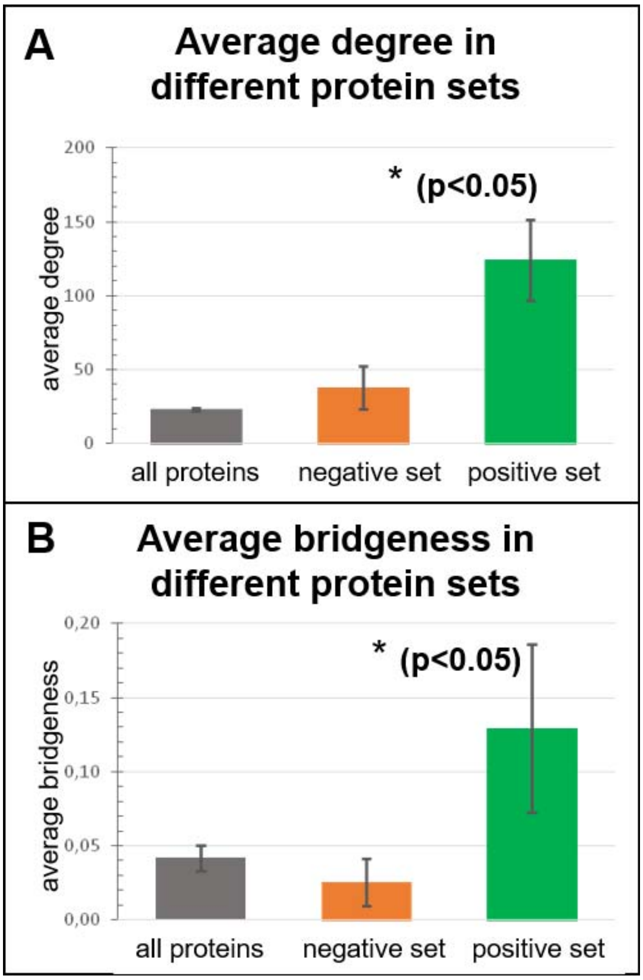

Supplementary Figure S2. Degree and bridgeness of all proteins, as well as the positive and negative training sets The figure shows the mean ± SD of the degree (Panel **A**) and the bridgeness (Panel **B**) values of all the 13 066 proteins as well as the 160 and 139 proteins of the positive and negative training sets, respectively. Using the giant component of the compartmentalized protein-protein interaction network (ComPPI)-derived human interactome having 151 889 interactions (all downloadable from here: http://translocatome.linkgroup.hu/download, 1) the degree and bridgeness were calculated by CytoScape (13) and by its network module determination plug-in, ModuLand (14), respectively. Panel A shows that the average degree is 23.2, 37.9 and 124.1 of all proteins, the negative and the positive training sets, respectively. The average degree of the positive set is significantly higher than the degree of the other two sets ($p<0.05$, Student's two tailed t-test). Panel B shows that the average bridgeness is 0.04, 0.03 and 0.13 of all proteins, the negative and positive training sets, respectively. The average bridgeness of the positive set is significantly higher than the bridgeness of the other two sets ($p<0.05$, Student's two-tailed t-test).

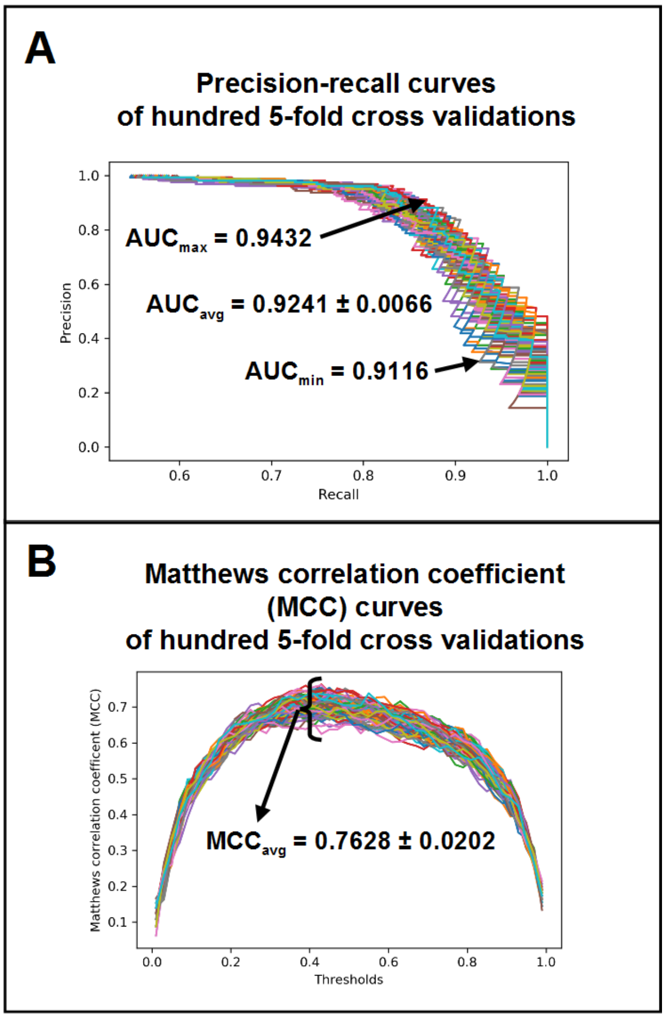

Supplementary Figure S3. Performance of the widely-used XGBoost machine learning method on the final feature set evaluated by precision-recall and Matthews correlation coefficient curves of hundred 5-fold cross validations

Each of the 100 different precision-recall (defined as Equations 1 and 2 of Supplementary Text S6) curves (panel A) and each of the 100 different Matthews correlation coefficient (MCC; defined as Equation 5 of Supplementary Text S6) curves (panel B) belong to a different 5 fold cross-validation run on the training set (containing 160 translocating and 139 non-translocating proteins). MCC values of the hundred 5-fold cross-validations were plotted as the function of threshold values dissecting the total range of MCC values to 50 equal segments. 5-fold cross validation runs were identical to those, whose receiver-operating characteristic (ROC) curves were shown on Figure 1C of the main text. ROC, precision-recall and MCC values are the generally suggested evaluation measures of machine learning methods (see e.g. in Refs. 12 and 15). In these 5-fold cross validation runs the well-established XGBoost machine learning method (3-5) used the final feature set (as shown on Table 1 of the main text) selected as described in the main text and Supplementary Text S6. The minimum, maximum and average of the area under the precision-recall curve values were 0.9116, 0.9432 and 0.9291 (±0.0066 standard deviation), respectively. The minimum, maximum and average of the maximum MCC values were 0.6627, 0.7628 and 0.7202 (±0.0202 standard deviation), respectively. MCC values range between -1.0 and +1.0, where all MCC values higher than zero mean better predictions than random choice, and MCC=1 means a perfect prediction (12,15,16).

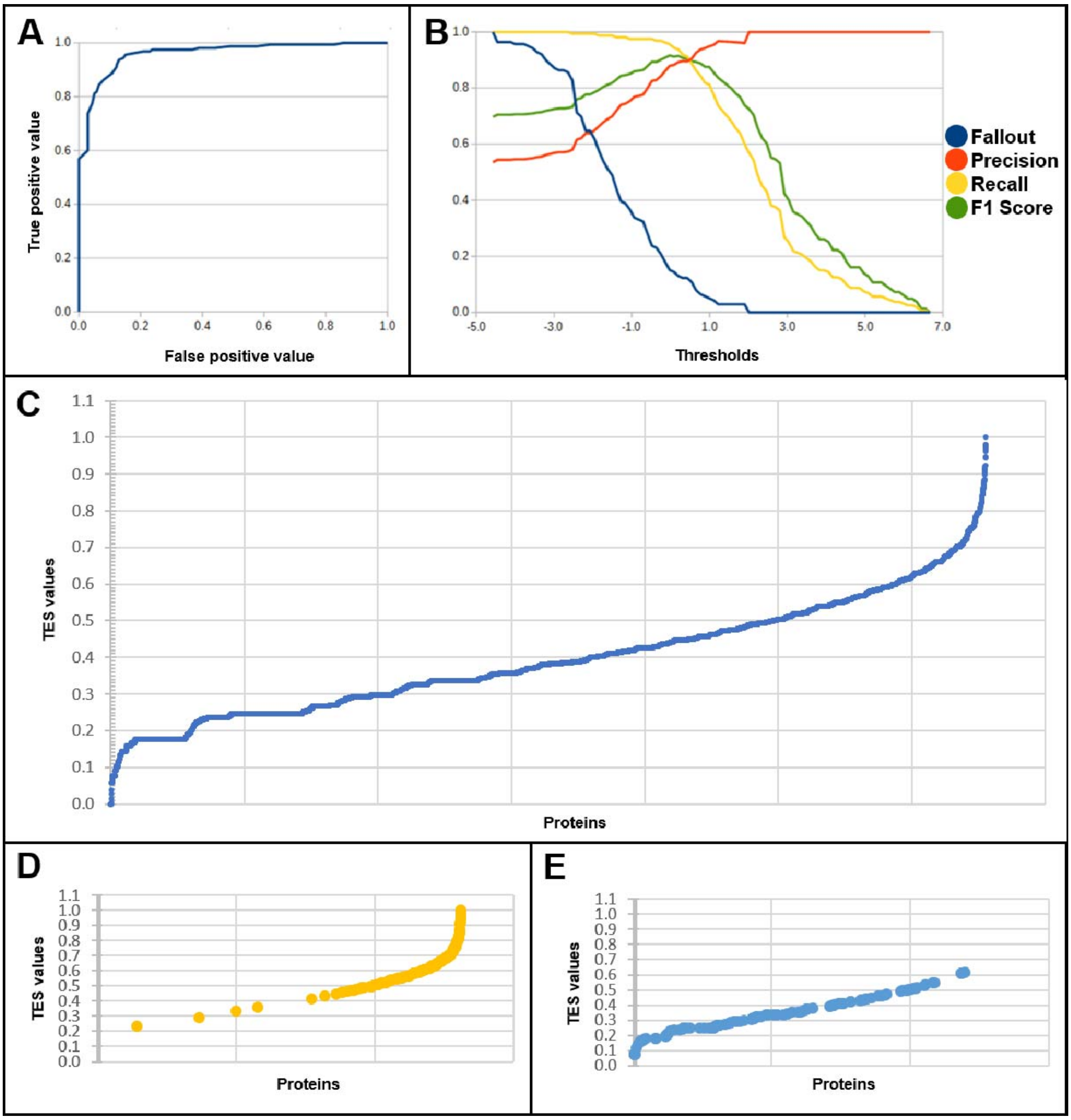

Supplementary Figure S4. Statistical parameters of the final prediction and translocation evidence-(TES)-scores Panel **A** shows the ROC curve for the prediction of the final model for the whole training set using the well-established, widely used XGBoost machine learning method (3-5, for more details see Supplementary Text S6). Panel **B** shows how the statistical parameters fallout, precision, recall and F1 score (see Equations 1 to 4 of Supplementary Text S6 and Supplementary Text S8) change their values as a function of the non-normalized Translocation Evidence Score (TES) values (for its detailed explanation see the main text). Panels **C, D** and **E** show the distribution of the TES values of all proteins, as well as those of the positive and negative training sets, respectively. Note that most of the positive training set entries have high TES values. On the contrary, most of the negative training set proteins have low TES values.

	A	B	C	D	E	F	G	H	I	J	K	L	M	N	O	P	Q	R	S	T	U	V	W	X
1	uniprot a	gene nar	protein r	referenc	localizat	localizat	localizat	transloca	transloca	structura	biologica	biologica	biologica	interacti	interacti	interacti	signaling	patholog	disease	exact dis	localization detection method			
2	Q16666	IFI16	Gamma-I	2,4E+07	{"major l	{"major l	N/A	Transloca	N/A	Nuclear l	N/A	N/A	N/A	HDAC fan	N/A	N/A	NOD-like	Localizat	Cancer	hepatoce	real time RT-PCR|Western blotting			
3	Q9GZX7	AICDA|AI	Single-st	24434356	{"major l	{"major l	N/A	Transloca	This tran	Nuclear l	N/A	cytidine	N/A	PKA	CTNNBL1	N/A	Mismatcl	Causes g	immunol	B cell lyn	confocal immunofluorescent microsco			
4	P18065	IGFBP2|I	Insulin-l	23435424	{"major l	{"major l	{"major l	Transloca	N/A	PSORT II	N/A	angioger	modulat	importin	N/A	N/A	EGFR tyrc	Has an o	Cancer	breast ca	immunoprecipitation|nuclear transpo			
5	Q16611	BAK1	Bcl-2 hor	2,4E+07	{"major l	{"major l	N/A	MTCH2 is	N/A	The hom	positive	N/A	N/A	myxoma	BCL2 (P10	N/A	Apoptosi	Induces	Cancer	N/A	N/A			
6	Q07812	BAX|BCL2	Apoptosi	1,5E+07	{"major l	{"major l	N/A	The JNK-r	N/A	Normally	binds to	positive	N/A	BCL2L11|	N/A	N/A	Apoptosi	N/A	Cancer	N/A	green fluorescent protein (GFP)-Bax fu			
7	O15392	BIRC5|IA	Baculovi	21900948	{"major l	{"major l	{"major l	Nuclear l	Released	N/A	cell divis	negative	negative	Centroso	Microtub	N/A	N/A	It orches	Cancer	Squamou	Immunostaining|TUNEL			
8	P17612	PRKACA	cAMP-de	1,9E+07	{"major l	{"major l	{"major l	N/A	N/A	N/A	N/A	N/A	N/A	N/A	N/A	N/A	N/A	N/A	N/A	N/A	N/A			
9	P04637	TP53|P53	Cellular t	24113167	{"major l	{"major l	{"major l	Transloca	E3 ligase	N/A	positive	regulatic	negative	DNA|Md	Mitochor	N/A	Apoptosi	Normally	Cancer	GBM	N/A			

Supplementary Figure S5. Data structure of manually curated translocating protein download files Pre-defined download option files are in comma separated format (.csv), which can be opened using a spreadsheet. The figure shows a representative section from the "Manually curated translocating proteins" file. Each row represents a protein, with its data next to it. Each column contains the type of information marked in the upper top row. These explanations of the abbreviations of data types are:

- **UniProt AC**: To identify every protein we use their UniProt accession number (6). You can find this number on the UniProt website (https://www.uniprot.org).
- **Gene name:** Here we use the UniProt (6) Gene name. As a default we use the primary gene name. Some of the entries has more than one gene names separated with a pipe symbol.
- **Protein name:** This is the full name of a protein from the UniProt database (6).
- **Reference:** List of the PubMed IDs of the articles that discusses the information added to the latter cells. If more than one, they are separated with a pipe symbol.
- **Localization A:** For every localization data we use a specific type of input (called subdocument). See the example of the IFI16 protein:

  > *{"major loc": ["nucleus (GO:0005634)"],"*
  > *minor loc":"nucleoplasm (GO:0005654)",*
  > *"comments": ["write your comments here"]}*

  Every localization item is stored according to the Gene Ontology naming convention (8,9). We use six major localizations: Cytoplasm, Nucleus, Mitochondrion, Extracellular space, Secretory-pathway, Membrane. "Minor loc" refers to a subcompartment of the six major localizations, if mentioned in the reference article. If there is any, comments are added to the "comments" section.

- **Localization B:** see at "Localization A"
- **Localization C:** see at "Localization A"
- **Translocation mechanism A-B:** Briefly summarizes data how the translocation occurs between localizations A and B.
- **Translocation mechanism B-C:** Briefly summarizes data how the translocation occurs between localizations B and C.
- **Structural information:** Briefly summarizes the structural properties of a given protein that are relevant for its translocation.
- **Biological process A:** List of the biological processes in localization A related to the protein stored according to the Gene Ontology naming convention (8,9), separated with the pipe symbol.
- **Biological process B:** see "Biological process A"
- **Biological process C:** see "Biological process A"
- **Interactions A:** The interacting protein names of corresponding entry in Localization A, specifically mentioned in the scientific paper cited in "Reference". If more than one, separated with the pipe symbol.
- **Interactions B**: The same as "Interactions A", but in Localization B.
- **Interactions C:** The same as "Interactions A", but in Localization C.
- **Signalling pathway**: The KEGG-based (17) naming convention of the signalling pathway associated with the protein. The following roles of the protein (often after posttranslational modification) are possible: inhibiting/blocking, enhancing, modifying, etc.

- **Pathological role:** If the cited scientific paper in "Reference" mentions anything about a pathological situation the translocation playing role in or leading to, it is to be marked here in as a pathophysiological factor (such as: tumorigenesis, angiogenesis, etc.).
- **Disease group**: Broad and general group of pathological states where the translocation was observed or occurs frequently (such as cancer).
- **Exact disease**: Exact disease, where the translocation was observed or occurs frequently.
- **Localization detection method**: The name of the biochemical method for observing the translocation (usually in a broader, general manner).

If any item of data is not available for the protein, the “N/A” marker is used.

	A	B	C	D	E	F	G	H	I	J
1	uniprot ac	gene nam	protein na	degree	betweenr	bridgenes	localizatic	translocation evidence score		
2	A1E959	ODAM	Odontoge	3.0	1.01e-06	2.0e-06	cytosol:0.7	0.6305		
3	A7KAX9	ARHGAP3:	Rho GTPas	24.0	2.606e-05	0.000446	cytosol:0.8	0.7187		
4	O00148	DDX39A	ATP-depe	36.0	0.0001111	0.611863	cytosol:0.9	0.633		
5	O00170	AIP	AH recept	28.0	0.0002386	0.015102	cytosol:0.9	0.6614		
6	O00213	APBB1	Amyloid b	71.0	0.0003435	0.328265	cytosol:0.9	0.702		
7	O00221	NFKBIE	NF-kappa-	50.0	5.157e-05	0.001217	cytosol:0.9	0.6614		
8	O00231	PSMD11	26S prote:	107.0	0.0003185	0.006785	cytosol:0.9	0.6338		
9	O00232	PSMD12	26S prote:	79.0	6.76e-05	0.009573	cytosol:0.9	0.7454		
[illegible]	[illegible]	[illegible]	[illegible]	[illegible]	[illegible]	[illegible]	[illegible]	[illegible]		

Supplementary Figure S6. Data structure of pre-defined download option files

Pre-defined download options are in comma separated format (.csv), which can be opened using a spreadsheet. The figure shows the representative view of the following files: “High-confidence translocating proteins”, “Low-confidence translocating proteins”, “Non-translocating proteins predicted by machine learning”, “Whole dataset of the Translocatome”. Note that “Manually curated non-translocating proteins” file differs from the above mentioned ones, because columns G and H are excluded. Please note that the high-confidence, low-confidence and non-translocating datasets do not contain the manually curated proteins, since the extensive data structure of the latter is available in different download formats (see Supplementary Figure S5). Each row represents a protein, with its data next to it. Each column contains the type of information marked in the upper top row. These explanations of the abbreviations of data types are:

- **UniProt AC:** To identify every protein we use their UniProt accession number (6). You can find this number on the UniProt website (https://www.uniprot.org).
- **Gene name:** Here we use the UniProt (6) Gene name. As a default we use the primary gene name. Some of the entries has more than one gene names separated with a pipe symbol.
- **Protein name:** This is the full name of a protein from the UniProt database (6).
- **Degree**: Number of interacting partners of the given protein, based on the human protein-protein interaction network constructed from the data of the ComPPI database (1). The measure was calculated using the CytoScape program (13).
- **Betweenness centrality:** the score represents the number of the shortest paths that pass through the protein. Higher betweenness centrality represents a higher ability to control the network, since more information passes through the protein. This centrality measure was not used in the machine learning process, since it did not characterize well the positive and negative training sets (data not shown). The measure was calculated using the CytoScape program (13).
- **Bridgeness:** Bridges are nodes which connect different network modules, i.e. large protein complexes in protein-protein interaction networks (18). Since translocating proteins often have different associating partners in their different locations they are often forming bridges in the protein-protein interaction network. The measure was calculated using the ModuLand CytoScape plug-in (14).
- **Localizations with localization score from ComPPI database** (excuded from “Manually curated non-translocating proteins”): The score gives the probability of a given protein to be found in a certain compartment, the number is imported from the ComPPI database (1).
- **Translocation evidence score** (excluded from “Manually curated non-translocating proteins”): The TES scores gives the likelihood of the protein to translocate as defined and discussed in detail in the main text.

	A	B	C	D	E	F	G	H	I	J	K	L	M	N	O
1	Protein_A	Naming_C	Synonyms	Taxonomy	Protein_B	Naming_C	Synonyms	Taxonomy	Interactio	Interactio	Interactio	Interactio	Interactio	Data_Source	
2	O75173	UniProtKE	O75173|A	9606	P01011	UniProtKE	P01011|SE	9606	0.9968744	two-hybri	HPRD	N/A	16099106	ComPPI_v1.1	
3	O75173	UniProtKE	O75173|A	9606	Q96GW7	UniProtKE	Q96GW7|	9606	0.9997399	in vitro(Ex	HPRD	N/A	10986281	ComPPI_v1.1	
4	O75173	UniProtKE	O75173|A	9606	P01009	UniProtKE	P01009|SE	9606	0.9991217	two-hybri	HPRD|HPI	N/A	16099106|	ComPPI_v1.1	
5	O75173	UniProtKE	O75173|A	9606	P00738	UniProtKE	P00738|HI	9606	0.9994462	two-hybri	HPRD	N/A	16099106	ComPPI_v1.1	
6	O75173	UniProtKE	O75173|A	9606	P16112	UniProtKE	P16112|A	9606	0.9987715	protease a	MatrixDB|	N/A	19744558|	ComPPI_v1.1	
7	O75173	UniProtKE	O75173|A	9606	P35625	UniProtKE	P35625|TI	9606	0.9996408	protease a	MatrixDB	N/A	19643179	ComPPI_v1.1	
8	O75173	UniProtKE	O75173|A	9606	Q05516	UniProtKE	Q05516|ZI	9606	0.24	physical ir	BioGRID|I	N/A	21988832|	ComPPI_v1.1	
9	O75173	UniProtKE	O75173|A	9606	Q96MU7	UniProtKE	Q96MU7|'	9606	0.0	physical ir	BioGRID|I	N/A	21988832|	ComPPI_v1.1	
10	O75173	UniProtKE	O75173|A	9606	Q15717	UniProtKE	Q15717|EI	9606	0.0	physical ir	BioGRID	N/A	19322201	ComPPI_v1.1	
11	O75173	UniProtKE	O75173|A	9606	P09958	UniProtKE	P09958|FU	9606	0.9973007	in vivo(Ex	HPRD	N/A	14744861	ComPPI_v1.1	
12	O75173	UniProtKE	O75173|A	9606	O60687	UniProtKE	O60687|SI	9606	0.9994452	two-hybri	HPRD|HPI	N/A	18718938|	ComPPI_v1.1	
13	P16112	UniProtKE	P16112|A	9606	P22894	UniProtKE	P22894|M	9606	0.9979114	in vitro(Ex	HPRD	N/A	8216228	ComPPI_v1.1	
14	P16112	UniProtKE	P16112|A	9606	P50281	UniProtKE	P50281|M	9606	0.9992210	in vivo(Ex	HPRD|HPI	N/A	11854269|	ComPPI_v1.1	
15	P16112	UniProtKE	P16112|A	9606	P51884	UniProtKE	P51884|LU	9606	0.9993775	physical ir	BioGRID|H	N/A	15505028|	ComPPI_v1.1	
16	P16112	UniProtKE	P16112|A	9606	Q9UHI8	UniProtKE	Q9UHI8|A	9606	0.9964003	in vitro(Ex	HPRD	N/A	12054629	ComPPI_v1.1	

Supplementary Figure S7. Data structure of the protein-protein interaction network downloadable file Pre-defined download options are in comma separated format (.csv), which can be opened using a spreadsheet. The figure shows the representative view of the following file: “Protein-protein interaction network of the Translocatome”. The following data columns (the entries are separated by |) can be seen:

- **Protein A:** UniProt (6) accession of interactor protein A.
- **Naming Convention A:** naming convention for the interactor protein A. Only Swiss-Prot entries were used, so the value is “UniProtKB/Swiss-Prot/P” for every entry.
- **Synonyms A:** list of the protein name synonyms for the interactor protein A
- **Taxonomy ID A:** the taxonomy ID of the interactor protein A, since all the protein are human, the ID is 9606 for every entry.
- **Protein B:** UniProt (6) accession of the interactor protein B
- **Naming Convention B:** naming convention for the interactor protein B. Only Swiss-Prot entries were used, so the value is “UniProtKB/Swiss-Prot/P” for every entry.
- **Synonyms B:** list of the protein name synonyms for the interactor protein B
- **Taxonomy ID B:** the taxonomy ID of the interactor protein B, since all the protein are human, the ID is 9606 for every entry.
- **Interaction Score:** the interaction score is defined as described in Ref. 1 and gives the probability of the interaction in a compartment dependent manner (Note that the interaction score is 0, if there was no localization information for one, or both of the interactors.)
- **Interaction Experimental System Type:** list of the experimental system types for the given interaction
- **Interaction Source Database:** list of the source databases for the given interaction as defined in the ComPPI (1) database.
- **Interaction PubMed ID:** list of the PubMed IDs for the sources of the given interaction.

Supplementary References

1. Veres,D.V., Gyurkó,D.M., Thaler,B., Szalay,K.Z., Fazekas,D., Korcsmáros,T. and Csermely,P. (2015) ComPPI: a cellular compartment-specific database for protein-protein interaction network analysis. *Nucleic Acids Res*., **43**, D485–D493.
2. Chen,C., Zabad,S., Liu,H., Wang,W. and Jeffery,C. (2018) MoonProt 2.0: an expansion and update of the moonlighting proteins database. *Nucleic Acids Res.,* **46**, D640−D644.
3. Friedman,J.H. (2001) Greedy function approximation: a gradient boosting machine. *Ann. Stat.,* **29**, 1189−1232.
4. Chen,T. and Guestrin,C. (2016) XGboost: A scalable tree boosting system. In Krishnapuram,B. and Shah,M. (ed.), Proceedings of the 22nd ACM SIGKDD International Conference on Knowledge Discovery and Data Mining. Association for Computing Machinery, New York, pp. 785−794.
5. Kerepesi,C., Daróczy,B., Sturm,Á., Vellai,T. and Benczúr,A. (2018) Prediction and characterization of human ageing-related proteins by using machine learning. *Sci. Rep.,* **8**, 4094.
6. UniProt Consortium. (2017). UniProt: the universal protein knowledgebase. *Nucleic Acids Res.,* **45**, D158−D169.
7. Thul,P.J., Åkesson,L., Wiking,M., Mahdessian,D., Geladaki,A., Ait Blal,H., Alm,T., Asplund,A., Björk,L., Breckels,L.M., *et al.* (2017) A subcellular map of the human proteome. *Science* **356**, eaal3321.
8. Ashburner,M., Ball,C.A., Blake,J.A., Botstein,D., Butler,H., Cherry,J.M., Davis,A.P., Dolinski,K., Dwight,S.S., Eppig,J.T., *et al.* (2000) Gene ontology: tool for the unification of biology. *Nat. Genet.,* **25**, 25–29.
9. The Gene Ontology Consortium. (2017) Expansion of the Gene Ontology knowledgebase and resources. *Nucleic Acids Res.,* **45**, D331–D338.
10. Freitas,A.A., Vasieva,O and de Magalhães,J.P. (2011) A data mining approach for classifying DNA repair genes into ageing-related or nonageing-related. *BMC Genomics,* **12**, 27.
11. Hanley,J.A. and McNeil,B.J. (1982) The meaning and use of the area under a receiver operating characteristic (ROC) curve. *Radiology*, **143**, 29−36.
12. Powers,D.M.W. (2011) Evaluation: From precision, recall and F-measure to ROC, informedness, markedness and correlation. *J. Machine Learning Technol.,* **2**, 37–63.
13. Shannon,P., Markiel,A., Ozier,O., Baliga,N.S., Wang,J.T., Ramage,D., Amin,N., Schwikowski,B. and Ideker,T. (2003) Cytoscape: a software environment for integrated models of biomolecular interaction networks. *Genome Res*., **13**, 2498–2504.
14. Szalay-Beko,M., Palotai,R., Szappanos,B., Kovács,I.A., Papp,B. and Csermely,P. (2012) ModuLand plug-in for Cytoscape: determination of hierarchical layers of overlapping network modules and community centrality. *Bioinformatics,* **28**, 2202−2204.
15. Chicco,D. (2017) Ten quick tips for machine learning in computational biology. *BioData Mining* **10**, 35.
16. Rahman,M.K., Rahman,M.S. (2017) CRISPRpred: A flexible and efficient tool for sgRNAs on-target activity prediction in CRISPR/Cas9 systems. *PLoS One* **12**, e0181943.
17. Kanehisa,M., Furumichi,M., Tanabe,M., Sato,Y. and Morishima,K. (2017) KEGG: new perspectives on genomes, pathways, diseases and drugs. *Nucleic Acids Res.,* **45**, D353–D361.
18. Kovács,I.A., Palotai,R., Szalay.M.S. and Csermely.P. (2010) Community landscapes: an integrative approach to determine overlapping network module hierarchy, identify key nodes and predict network dynamics. *PLoS One,* **2**, e12528.
